# Proteomics of yeast telomerase identified Cdc48-Npl4-Ufd1 and Ufd4 as regulators of Est1 and telomere length

**DOI:** 10.1038/ncomms9290

**Published:** 2015-09-14

**Authors:** Kah-Wai Lin, Karin R. McDonald, Amanda J. Guise, Angela Chan, Ileana M. Cristea, Virginia A. Zakian

**Affiliations:** 1 Department of Molecular Biology, Lewis Thomas Laboratory, Princeton University, Washington Road, 08544 Princeton, New Jersey, USA

## Abstract

Almost 400 genes affect yeast telomere length, including *Est1*, which is critical for recruitment and activation of telomerase. Here we use mass spectrometry to identify novel telomerase regulators by their co-purification with the telomerase holoenzyme. In addition to all known subunits, over 100 proteins are telomerase associated, including all three subunits of the essential Cdc48-Npl4-Ufd1 complex as well as three E3 ubiquitin ligases. The Cdc48 complex is evolutionarily conserved and targets ubiquitinated proteins for degradation. Est1 levels are ∼40-fold higher in cells with reduced Cdc48, yet, paradoxically, telomeres are shorter. Furthermore, Est1 is ubiquitinated and its cell cycle-regulated abundance is lost in Cdc48-deficient cells. Deletion of the telomerase-associated E3 ligase, Ufd4, in *cdc48-3* cells further increases Est1 abundance but suppresses the telomere length phenotype of the single mutant. These data argue that, in concert with Ufd4, the Cdc48 complex regulates telomerase by controlling the level and activity of Est1.

## Abstract (web summary)

Regulating telomere length and telomerase activity are critical biological processes implicated in ageing and cancer. Here the authors use mass spectrometry to identify the Cdc48-Npl4-Ufd1 complex, which targets proteins for degradation, as a novel regulator of the yeast telomerase Est1.

## Graphical abstract

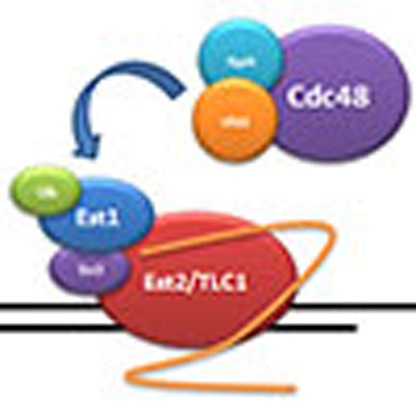

Owing to the properties of conventional DNA polymerases, the very ends of linear DNA molecules require a special replication mechanism, which in almost all eukaryotes is provided by telomerase, a telomere-dedicated reverse transcriptase. The catalytic core of telomerase consists of the reverse transcriptase (hTERT, humans; Est2, budding yeast) and a templating RNA (hTR, humans; TLC1, budding yeast). In addition, the telomerase holoenzyme contains accessory proteins that are essential for the stability and/or activity of the telomerase holoenzyme *in vivo*.

Est1 is the best conserved of the telomerase accessory proteins, present from yeasts to humans. In budding and fission yeasts, Est1 is essential for telomerase-mediated telomere maintenance with roles in both recruitment and activation of telomerase^1^,^2^,^3^. The *Saccharomyces cerevisiae* telomerase accessory protein Est3 (refs 4, 5), which is also essential for *in-vivo* telomerase activity, is found only in budding yeasts. However, structural predictions suggest that it is similar to the mammalian TPP1 (refs 4, 5), a telomere structural protein that increases telomerase processivity^6^,^7^. In budding yeast, Est3 interacts directly with both Est1 (ref. 8) and Est2 (refs 9, 10), and its association with Est1 is required for its recruitment to telomeres *in vivo*^8^.

Telomerase activity is tightly regulated. Part of this regulation is at the level of abundance, as telomerase is either absent (most human somatic cells)^11^ or present in very low amounts, fewer than one complex per telomere (budding and fission yeasts)^1^,^12^,^13^. Even in human stem cells that express telomerase, its activity is low, as heterozygous mutations that reduce the abundance of a telomerase component or telomere structural protein can cause premature death as a result of stem cell failure (reviewed in refs 14, 15). Likewise, telomerase RNA is haplo-insufficient for maintenance of telomere length in both budding^13^ and fission yeasts, as is the fission yeast Est1 (Webb and Zakian, in preparation). Telomerase is upregulated in ∼85% of human tumours^15^ and its expression promotes tumorigenesis^16^. Thus, understanding telomerase regulation is relevant to ageing and cancer.

In budding and fission yeast, telomerase action is cell cycle regulated, occurring only in late S/G2 phase^17^,^18^. In budding yeast, part of this regulation is due to the cell cycle-regulated expression of Est1, which is low in G1 phase (∼20 molecules per cell) and high at the end of the cell cycle (∼110 molecules per cell) when telomerase is active^1^,^19^. Although Est1 is the only core subunit of *S. cerevisiae* telomerase whose abundance is cell cycle regulated, given that it is required to recruit Est3 into the holoenzyme and to telomeres^8^, Est3 action is also cell cycle regulated. Although Est1 regulation is not sufficient to restrict telomerase to a short window of the cell cycle^20^, its biology is key for understanding cell cycle regulation of telomerase.

In budding and fission yeasts, genetics has been extremely successful at identifying a large number of genes whose mutation affects telomere length (reviewed in refs 21, 22). However, for most of these genes, it is not known whether they affect telomeres directly. We reasoned that mass spectrometry (MS) analysis of telomerase might identify proteins with direct effects on telomerase. Indeed, MS has been extremely useful for identifying novel telomerase components and regulators from both ciliates^23^,^24^,^25^ and human cultured cells^26^,^27^,^28^. However, the very low abundance of yeast telomerase has hampered attempts to use MS, to identify proteins that co-purify with telomerase.

We describe methods to purify active telomerase from budding yeast and to identify interacting proteins by MS. This approach identified multiple candidates for novel telomerase regulatory proteins, including the three subunits of a Cdc48 complex, Cdc48, Npl4 and Ufd1. Cdc48, an AAA ATPase, is an evolutionarily conserved protein that is the catalytic subunit of several multi-protein complexes that contain ubiquitin receptors, such as Npl4 and Ufd1 (reviewed in ref. 29). These complexes act as segregases that recognize and remove ubiquitinated proteins from multi-protein complexes^30^,^31^,^32^. From yeasts to humans, Cdc48 complexes are implicated in a plethora of diverse nuclear and cytoplasmic processes, including cell cycle progression, membrane fusion, repair of double-strand breaks, termination of DNA replication, modification of transcription factors, postmitotic reassembly of the nuclear envelope and export of proteins from the endoplasmic reticulum and mitochondria (reviewed in ref. 33). However, Cdc48 has not been linked previously to telomerase. The diverse phenotypes associated with loss of Cdc48 are mostly due to its role in promoting proteasome-mediated degradation, where it acts at a step after ubiquitination and before degradation of target proteins^34^,^35^. Mutations of its human homologue, p97 or VCP (valosin-containing protein), cause a dominant multi-organ and progressive degenerative syndrome of the muscles, bone and brain, called VCP disease^36^.

Here we report a previously unrecognized mechanism of telomerase regulation in which the Cdc48-Npl4-Ufd1 complex affects Est1 abundance, activity and cell cycle regulation, as well as telomere length. Moreover, deletion of a telomerase-associated E3 ubiquitin ligase (E3 Ub), Ufd4, which has also not been linked previously to telomeres, results in long telomeres, while its deletion from *cdc48-3* cells suppresses the short telomere length phenotype of *cdc48-3* cells. These data suggest that Ufd4 ubiquitinates Est1 and this modified Est1 is removed from the holoenzyme by the Cdc48 complex. As the Cdc48 complex and Est1 are both highly conserved, these data may be relevant to telomerase regulation in humans.

## Results

### Generation of a telomerase-overexpressing strain

**Figure 1:**
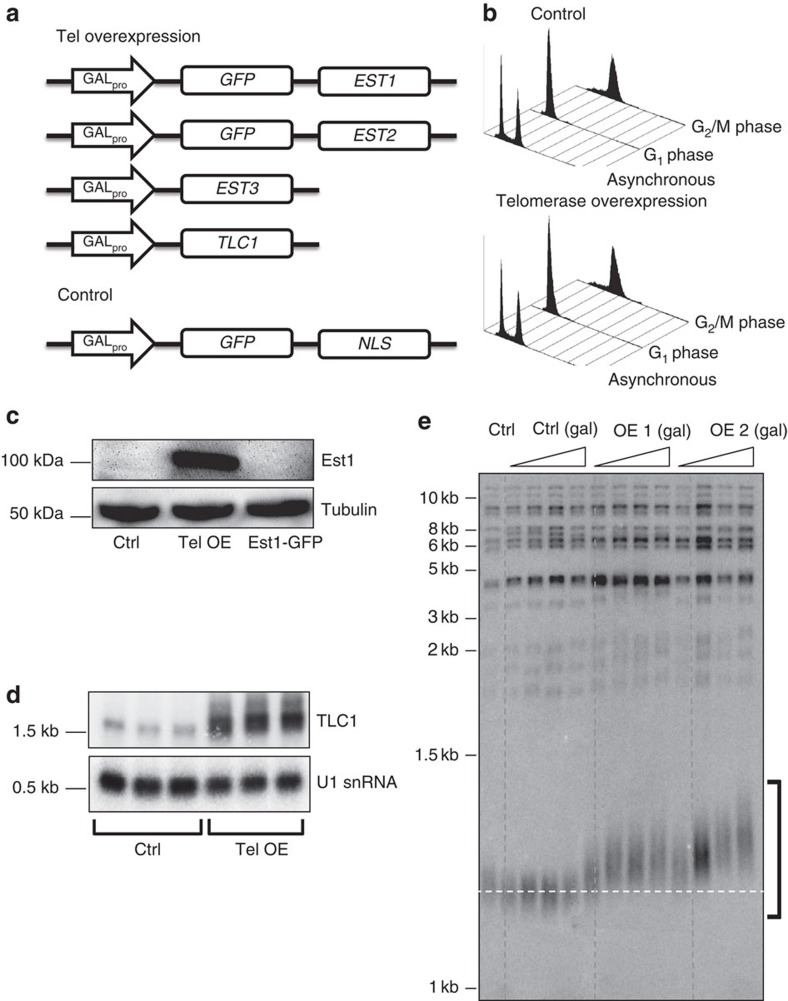
Generation and characterization of strains for purification of telomerase. (**a**) In the telomerase OE strain, each of the four essential telomerase subunits, Est1, Est2, Est3 and TLC1 RNA, was expressed from the galactose-inducible *GAL1* promoter as the only form of the gene product in the strain. Est1 and Est2 were expressed as GFP fusions. The control for the telomerase OE strain contained a fusion gene, *GAL1-GFP*-NLS in which the nuclear localization signal (NLS) from the SV40 T-antigen was fused to GFP. (**b**) FACS analyses of both the control (top) and telomerase OE strains (bottom) as asynchronous cultures or arrested in G1 or G2/M phase by α-factor and nocodazole, respectively. (**c**) Western blot analysis using an anti-GFP antibody readily detected Est1 in galactose-grown telomerase OE cells but not in galactose-grown control strain (Ctrl) nor from glucose-grown cells expressing Est1-GFP from the *EST1* promoter. The full lane view of this blot is shown in Supplementary Fig. 1C. (**d**) Northern blot analysis was carried out on three isolates from both the control and telomerase OE strain growing in galactose media. The northern blot was probed for TLC1 RNA and for U1 snRNA. Quantification by ImageQuant TL (GE Healthcare) indicated that there was 10–12 times more TLC1 RNA in the telomerase OE cells compared with the control. The full lane view of this blot is shown in Supplementary Fig. 1D. (**e**) OE of telomerase results in telomere lengthening. Southern analysis of telomere length in two independent isolates of telomerase OE cells (OE1 and OE2) growing in galactose for four successive re-streaks compared with control cells grown in the same way (Ctrl(gal)). Lane labelled Ctrl is from glucose-grown control cells. DNA was digested with XhoI. The blot was probed with pCT300, which detects TG_1–3_ telomeric repeats. Size markers are in kbp. Bracket indicates terminal restriction fragments from Y'-bearing telomeres. Here and in subsequent figures, horizontal dashed line marks mean telomere length in control cells.

The low abundance of *S. cerevisiae* telomerase has thwarted previous attempts to study it by MS. To circumvent this problem, we generated a strain that overexpressed (OE) each of the four essential telomerase subunits: TLC1 RNA, Est1, Est2 and Est3 (hereafter OE strain). The chromosomal copy of the gene for each component was put under the control of the strong and inducible *GAL1* promoter. To aid in immunopurification of the holoenzyme, Est1 and Est2 were expressed as green fluorescent protein (GFP) fusions (Fig. 1a). A strain with a *GAL1*-regulated nuclear-localized GFP was used as a specificity control as it allowed elimination of proteins with nonspecific associations to the resin or GFP tag (Fig. 1a, Control). The telomerase OE strain had normal viability, growth rate and cell cycle progression (Fig. 1b).

Western blot analysis using an anti-GFP antibody readily detected Est1 and Est2 in the OE strain (Fig. 1c and Supplementary Fig. 1A). However, the level of overexpression of these proteins could not be determined, because neither Est1-GFP nor Est2-GFP expressed from its endogenous promoter was detectable by western blotting (Fig. 1c and Supplementary Fig. 1C). Therefore, we used the abundance of TLC1 RNA in wild-type (WT) versus telomerase OE strain to estimate the extent of overexpression (Fig. 1d and Supplementary Fig. 1D). Northern analysis indicated approximately ten times more telomerase RNA in the OE strain than in WT cells (Fig. 1d and Supplementary Fig. 1D). As TLC1 is the subunit present in the lowest number per cell, this value is a good approximation of the maximum level of holoenzyme overexpression^1^,^8^,^13^.

To determine whether the OE telomerase was active, we used Southern analysis to determine telomere length in two independent isolates of the OE strain (Fig. 1e). Telomeres in the two OE isolates progressively lengthened over four successive restreaks. By restreak four, telomeres were ∼80–130 bp longer than the ∼300 bp in the galactose grown control. Thus, the OE telomerase is active.

### Proteomic profiling of purified telomerase

GFP-tagged telomerase components Est1 and Est2 were immuno-affinity purified using high-titre polyclonal rabbit anti-GFP antibodies and extraction conditions that optimized telomerase recovery^37^. As telomerase activity is regulated during the cell cycle, telomerase was purified from cells arrested in either late G1 phase by treatment with α-factor (telomerase inactive) or in G2/M phase by treatment with nocodazole (telomerase active) (Fig. 1b and Supplementary Fig. 1A)^17^,^18^. Lysates were treated (or not) with DNase I to distinguish proteins with DNA-dependent associations and resolved by SDS–polyacrylamide gel electrophoresis (SDS–PAGE) before MS analysis (Supplementary Fig. 1B).

To determine the specificity of individual protein interactions, we used the SAINT (Significance Analysis of INTeractome) algorithm to calculate specificity scores by comparing proteins that co-isolated with telomerase to GFP controls^38^. The SAINT algorithm employs a probabilistic scoring method incorporating the number of spectra detected in both experimental and control isolations, frequency of detection across biological replicates and protein length. We adopted a stringent SAINT score cutoff of 0.8, which retained only the most prominent interactions (top ∼5%, Supplementary Data 1). Using these criteria, a combined total of 115 proteins were identified in the G1 and G2/M isolations (89 with G1 phase telomerase and 72 with G2/M phase telomerase; Supplementary Data 1). Of these, 32% were telomerase associated after DNase I treatment (37 proteins in both G1 and in G2/M phases; these proteins are in bold and italics in Supplementary Data 1).

### Isolation method identified all known telomerase subunits

As expected, given the isolation strategy, GFP-tagged Est1 and Est2 were both identified with high confidence from immuno-isolates, as demonstrated by very high sequence coverage (Est1: 78%; Est2: 70%) and high spectral counts (Supplementary Data 1). Likewise, the other essential telomerase protein, Est3, which was not GFP tagged, was present with high confidence in the immuno-isolates (Supplementary Data 1). TLC1 RNA has an Sm protein-binding site near the 3′-end of the molecule, whose mutation leads to reduced TLC1 levels and short telomeres, and two of the seven Sm proteins, Smd1 and Smd3, bind TLC1 RNA *in vivo*^39^. All seven Sm proteins (Smd1, Smd2, Smd3, Smb1, Sme1, Smx2 and Smx3) were identified with high specificity scores; Sme1 was the only one that was not present in both G1 and G2/M phase telomerase. With the exception of Smx2 and Smx3, all of these associations were DNase I resistant (Supplementary Data 1). In addition, 32 of the G1 and G2/M telomerase-associated proteins had been linked previously to telomere length or telomerase regulation (Supplementary Data 1). Thus, all known telomerase subunits and 22 proteins with reported links to telomere biology were present in the immuno-isolates, representing ∼30% of the 115 proteins identified. However, the majority of the associated proteins that co-purified with Est1/Est2 had not been linked previously to telomerase and are candidates for new telomerase regulators.

### Analysis of the telomerase interactome

**Figure 2:**
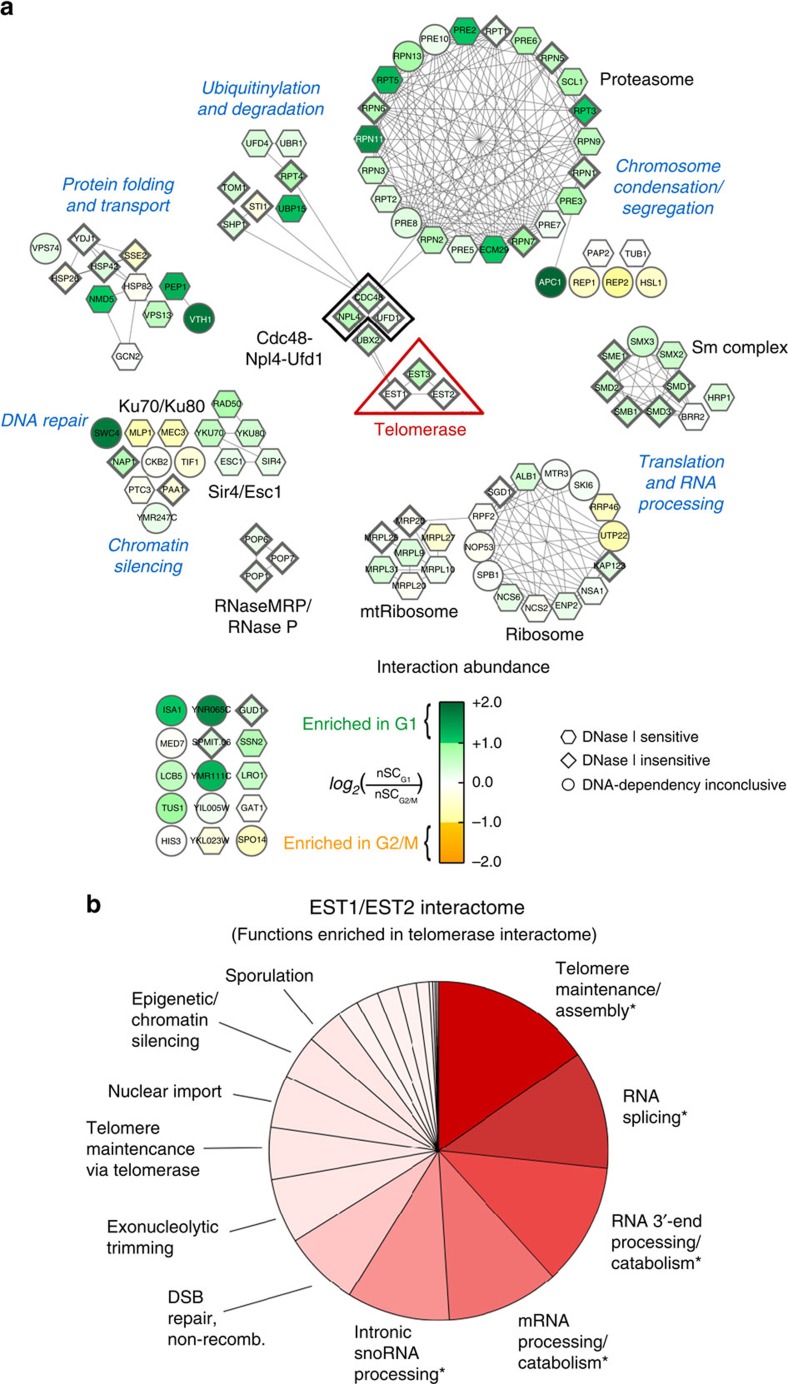
Functional protein interaction network of telomerase. (**a**) Cell cycle-dependent changes in protein associations of Est1/Est2. Node colours indicate relative changes in abundance of associations of individual proteins with telomerase during G1 or G2/M isolation, as determined by label-free normalized spectrum count comparison (green: enriched in G1; orange, enriched in G2/M). Prominent biological functions are represented in the network. Node shapes indicate the dependence of individual interactions on DNA (hexagons, DNA-dependent; diamonds, DNA-independent; circles, DNA-dependency inconclusive). Edges indicate functional connections among nodes curated by the STRING database. Surprisingly, Est3 was more abundant in G1 than in G2/M phase telomerase. (**b**) Gene Ontology enrichment analysis for high specificity (SAINT⩾0.8) telomerase interactions present in G1 and G2/M. Functional enrichment comparisons were performed against a whole *S. cerevisiae* genome background gene list; wedge sizes represent relative DAVID group-enrichment scores (min=0.03/max=2.63); * and bold represents significantly enriched categories (*P*<0.05, modified Fisher's exact test (EASE)^44^); colour grading represents relative enrichment over the background genome.

We used information from STRING-db^40^ and Cytoscape^41^, to generate a functional protein interaction network (Fig. 2a). Proteins were clustered into functional protein complexes and groupings using *Saccharomyces* Genome Database (SGD) annotations and STRING connectivity (Table 1 and Fig. 2a).

Multiple proteins involved in proteolysis were detected in both G1 and G2/M telomerase, although they were often enriched in G1 isolations (Table 1). This subset includes Cdc48, Npl4 and Ufd1, which act in a complex to target ubiquitinated proteins for proteosomal degradation (Fig. 2a and Supplementary Fig. 2). The Cdc48 cofactor Shp1 was also more abundant in G1 phase telomerase isolations, as were 22 of the 32 subunits of the proteasome (Fig. 2a and Table 1). Three E3 Ub ligases, Tom1, Ubr1 and Ufd4, as well as the deubiquitinase, Ubp15, were also telomerase associated with increased interaction abundance in G1 phase (Fig. 2a and Table 1).

In addition to the Cdc48 and proteasome complexes, other multi-subunit complexes were telomerase associated. For example, Pop1, Pop6 and Pop7 are components of both RNase MRP and nuclear RNase P. Both complexes are involved in RNA processing^42^.

Among the most prominent associations with known telomere functions were Yku70 and Yku80, which form a heterodimer that binds telomeres and telomerase RNA (reviewed in ref. 21). However, the association of both proteins was DNase sensitive (Fig. 2a, hexagon-shaped nodes), suggesting that the Yku70/80 complex is not part of the holoenzyme. Similarly, the telomerase association of Hsp82, a protein implicated in regulating the DNA-binding activity of Cdc13 (ref. 43), was DNase sensitive.

Using the DAVID Bioinformatics Database^44^, we determined whether specific functional classes were more highly represented in the telomerase isolations than in the yeast genome as a whole. This functional annotation demonstrated significant (*P*<0.05, modified Fisher's exact test (EASE)^44^) enrichment of proteins in telomere maintenance/assembly, RNA splicing, RNA 3′-end processing/catabolism, messenger RNA processing/catabolism, proteolysis, ubiquitin-dependent processes and small nucleolar RNA processing (Fig. 2b).

### The Cdc48 complex affects telomere length

To determine whether novel telomerase-associated protein had effects on telomere maintenance, we started with the Cdc48 complex as all three of its subunits, Cdc48, Npl4 and Ufd1, were among the most prominent telomerase-associated proteins with SAINT scores of 1 in both G1 and G2/M phase, and the association of each subunit was DNase resistant. In addition, the recovered peptides had high amino acid sequence coverage (an average of 76% for Cdc48, 36% for Npl4 and 37% for Ufd1).

If the association of the Cdc48 complex with telomerase is functionally important, we expect its loss to affect telomere length. As each of the three subunits is essential, we examined telomere length as well as other phenotypes in strains with temperature-sensitive alleles, *cdc48-3*, *npl4-1* and *ufd1-2* (ref. 45). Each of these strains arrests at mitosis when grown at 37 °C, the restrictive temperature for all three alleles. Although *cdc48-3* cells are viable at 25 °C (permissive temperature) and 30 °C (semi-permissive temperatures), proteolytic defects of are evident at both temperatures^45^.

**Figure 3:**
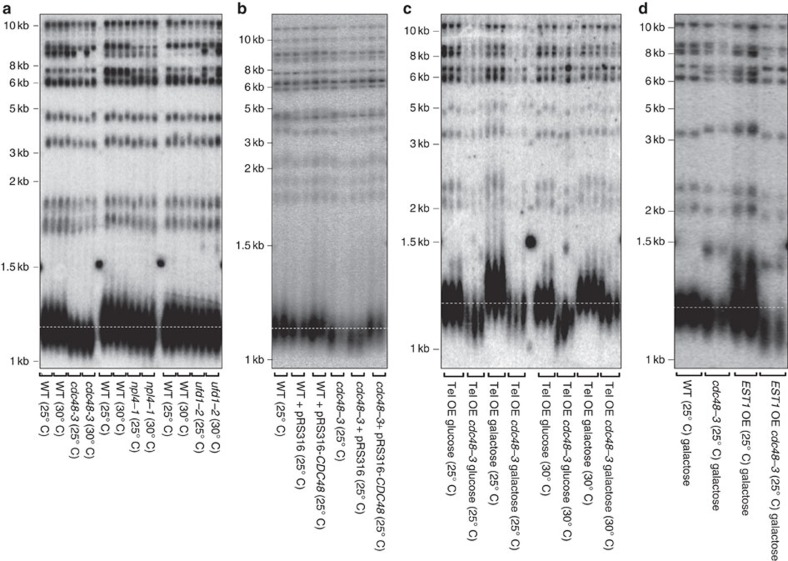
Telomeres are shorter in *cdc48-3* and *npl4-1* cells than in WT cells. (**a**) Southern analysis of DNA from two independent colonies of *cdc48-3*, *npl4-1*, and *ufd1-2* cells and their respective (WT) isogenic controls was digested with XhoI and analysed by Southern blotting. Temperatures at which cells were grown are indicated. (**b**) Telomere length of WT or *cdc48-3* cells carrying a centromere plasmid with (pRS316-*CDC48*) or without the *CDC48* gene (pRS316) was determined as in **a**. Symbols are as in **a**. (**c**) Southern analysis showed *cdc48-3* cells have shorter telomere length in telomerase OE cells, as compared with their WT counterpart, at both permissive and semi-permissive temperatures. Each lane is an independent isolate. (**d**) Southern analysis of telomere length in Est1 OE *cdc48-3* and WT cells.

DNA prepared from the mutant strains grown at both 25° and 30 °C was digested with restriction enzymes and subjected to Southern blot analysis using a probe for telomeric DNA (Fig. 3a). Telomeres in *cdc48-3* cells were about 40 bp shorter than an otherwise isogenic WT strain at both 25 °C and 30 °C. This short telomere phenotype was rescued by introducing a centromere plasmid containing *CDC48* (pRS316-*CDC48* (ref. 46 and Fig. 3b). Similarly, a *cdc48-3* telomerase OE strain had shorter telomeres than WT cells at both 25 °C and 30 °C (Fig. 3c). Thus, the effects of the *cdc48-3* mutation on telomere length cannot be rescued by overexpression of telomerase. In addition, *npl4-1* cells had telomeres that were modestly shorter than the WT isogenic control at both 25 °C and 30 °C, whereas *ufd1-2* cells had WT length telomeres (Fig. 3a). We focused additional experiments on *cdc48-3* cells as this mutant had the largest effect on telomere length.

### Telomeric silencing is modestly reduced in *cdc48-3* cells

To provide more evidence for an effect of the Cdc48 complex on telomeres, we examined the level of telomere position effect (TPE) in mutant versus WT cells. TPE refers to the reversible repression of telomeric gene transcription^47^. In otherwise isogenic strains, cells with long telomeres have more TPE than cells with short telomeres. To monitor TPE, we integrated the *URA3* gene adjacent to the left telomere of chromosome VII. Silencing of *URA3* allows growth in the presence of 5-fluoroorotic acid (5-FOA). Consistent with the telomere length data, *cdc48-3* cells had ∼5 × lower TPE than WT cells, whereas TPE levels in *npl4-1* and *ufd1-2* cells were the same as WT (Supplementary Fig. 3IA,B,C). TPE was also lower in *cdc48-3* cells that overexpress telomerase (Supplementary Fig. 3IIA). The TPE data provide additional support for a telomeric role for the Cdc48 complex.

### Cdc48 complex regulates Est1 abundance

**Figure 4:**
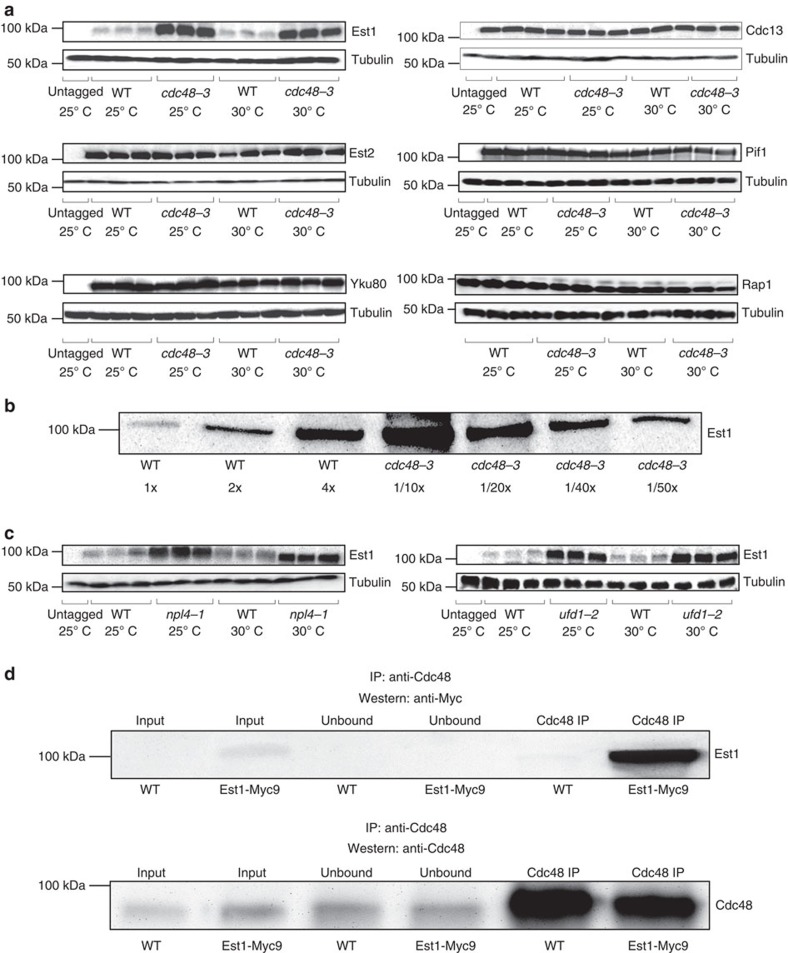
Est1 physically interacts with Cdc48 and its abundance is increased in *cdc48-3*, *npl4-1* and *ufd1-2* cells. (**a**) Proteins were prepared from three independent isolates of asynchronous *cdc48-3* cells grown at 25 °C (permissive temperature) and 30 °C (semi-permissive temperature) or from otherwise isogenic control cells and subjected to SDS–PAGE and western blot analysis. Est1, Est2, Cdc13, Yku80 and Pif1 were visualized with mouse anti-Myc antibodies (Clontech); Rap1 was visualized with rabbit anti-Rap1 antisera (Conrad *et al*.^48^); α-tubulin was visualized with rat anti-α-tubulin antibodies (Abcam). (**b**) Quantification of Est1 levels in WT versus *cdc48-3* cells. Extracts were diluted as indicated (or not diluted, 1 × ) and subjected to SDS–PAGE and western blot analysis. Est1 was ∼40-fold more abundant in *cdc48-3* compared with WT cells. (**c**) Est1 levels were elevated in both *npl4-1* and *ufd1-2* cells compared with their WT counterparts. Methods are the same as in **a**. (**d**) Extract of proteins from untagged WT cells or WT cells expressing Est1-Myc9 (input) was immunoprecipitated with goat anti-Cdc48 polyclonal antibodies (Abgent) (Cdc48 IP) and the samples analysed by western blotting with anti-Myc antibodies (Clontech). Supernatants recovered before and after immunoprecipitation were used as input and unbound controls, respectively (top panel). The same samples were analysed by western blotting using anti-Cdc48 antibodies (Abgent) (bottom panel). See Supplementary Fig. 4 for full lane view of these data.

Given that the Cdc48 complex promotes proteasome-mediated degradation of its target proteins, we considered that its effects on telomere length and TPE might reflect altered abundance of one or more telomere proteins. To test this possibility, we used western analysis to determine the levels of five Myc-tagged proteins, Est1, Est2, Cdc13, Yku80 and Pif1, and an untagged protein, Rap1, which was detected using a polyclonal antiserum^48^. Est1 and Est2 are core telomerase subunits, Cdc13 and Yku80 are telomere-binding proteins that affect both telomere structure and recruitment of telomerase, Rap1 is a telomere-binding protein that is essential for telomere protection and TPE, and Pif1 is a DNA helicase that removes telomerase from DNA ends (reviewed in ref. 21). We compared the abundance of each protein in WT and *cdc48-3* cells grown at both 25 °C and 30 °C using α-tubulin as a loading control (Fig. 4 and Supplementary Fig. 4).

Western blot analysis revealed that Est2, Cdc13, Yku80, Pif1 and Rap1 were expressed at about the same levels in *cdc48-3* and WT cells, while Est1 levels were much higher in *cdc48-3* cells (Fig. 4a and Supplementary Fig. 4A). This difference was evident in *cdc48-3* cells grown at both 25 °C and 30 °C compared with WT cells grown at the same temperatures. To estimate the increase in Est1 levels in *cdc48-3* cells, we did western blot analysis on a dilution series of the extract from both WT and mutant cells. As the western signal in the undiluted WT extract was comparable to that in a 40-fold dilution of the *cdc48-3* extract, Est1 abundance was about 40 times higher in mutant cells (Fig. 4b and Supplementary Fig. 4B). Likewise, Est1 was more abundant in both 25 °C- and 30 °C-grown *npl4-1* and *ufd1-2* cells compared with WT (Fig. 4c and Supplementary Fig. 4C). In *ufd1-2* cells, the Est1 increase was more evident at the more restrictive temperature of 30 °C than at 25 °C. As each mutation affected Est1 levels, the short telomeres in *cdc48-3* cells are probably due to reduced Cdc48 complex and the more modest effects of the other mutations probably reflect differences in the strengths of the three conditional alleles.

The Cdc48 complex was identified by MS as co-purifying with the Est1-GFP and Est2-GFP, and western blot analysis showed that Est1 abundance was Cdc48 dependent (Fig. 4a and Supplementary Fig. 4A). If the Cdc48 complex interacts specifically with Est1, Est1 should also be present in a Cdc48 immunoprecipitate. To test this possibility, we immunoprecipitated Cdc48 with a goat anti-Cdc48 polyclonal antiserum (Abgent) in cells expressing Est1-Myc. The low-abundance Est1 was readily detected in anti-Cdc48 immunoprecipitates (Fig. 4d and Supplementary Fig. 4D), confirming the Est1-Cdc48 interaction.

### Short *cdc48-3* telomeres are not due to limited Est3

Given the activation function of Est1, *a priori* one expects telomere lengthening rather than the observed telomere shortening when Est1 is OE, as it was in *cdc48-3* cells. To test this possibility, we OE Est1 by placing its transcription under the control of the galactose-inducible *GAL1* promoter. As reported previously^49^, overexpression of Est1 in our WT background resulted in telomere lengthening (∼80 bps) (Fig. 3d). However, telomeres were even shorter in *cdc48-3* cells expressing Est1 from the *GAL1* promoter than in *cdc48-3* cells alone (25 °C; Fig. 3d). Thus, the positive impact of OE Est1 on telomere length requires the Cdc48 complex.

Est3 interacts directly with Est1 and requires it for telomere binding^8^. Thus, another possibility for short telomeres in *cdc48-3* cells is that the very high levels of Est1 sequester Est3 from telomeres. If this model is correct, overexpression of Est3 in *cdc48-3* cells might restore telomeres to a more WT-like length. However, Est3 overexpression had little or no effect on telomere length in *cdc48-3* or WT cells (Supplementary Fig. 5), suggesting that telomere shortening in *cdc48-3* cells is not a consequence of cells being limited for Est3.

### Est1 is ubiquitinated in *cdc48-3* cells

**Figure 5:**
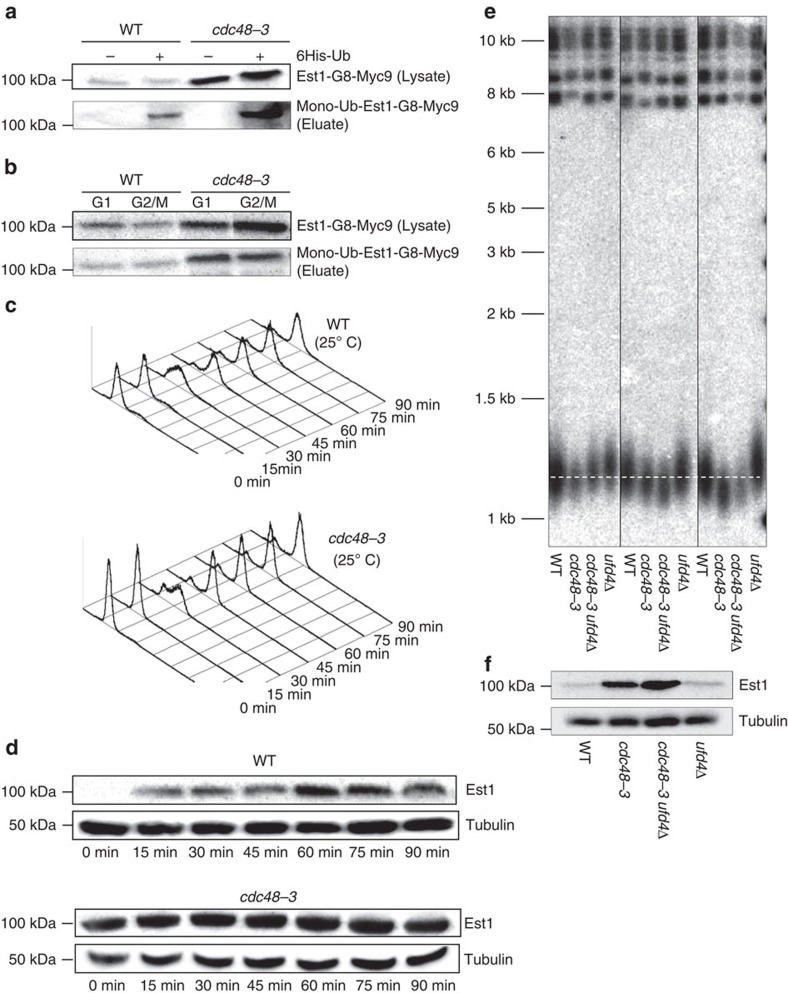
Ubiquitination of Est1 is increased and cell cycle-regulated abundance of Est1 is lost in *cdc48-3* cells. (**a**) WT and *cdc48-3* cells with Est1-G8-Myc9 were transformed with a plasmid encoding 6His-ubiquitin (‘+', 6His-Ub induction). In both the lysate and eluate, there was 10X more extract from WT cells than from cdc48-3 cells. Est1 was detected in both strains using anti-Myc western blotting in whole-cell lysates and after elution from Ni-NTA agarose, which purifies ubiquitinated proteins. See Supplementary Fig. 6A for full lane view of these data. (**b**) Samples were arrested in late G1 phase with α-factor (G1) and in G2/M phase with nocodazole (G2/M) and Est1 ubiquitination was detected using western analysis. See Supplementary Fig. 6C for full lane view of these data. (**c**) FACS profiles of WT (top) and *cdc48-3* (bottom) cells grown at 25 °C. Cells were arrested in late G1 phase with α-factor (0 min time point), removed from α-factor and allowed to proceed synchronously through the cell cycle. Samples were removed for FACS and western analysis (**d**) at the indicated time points with anti-Myc. The same membrane was probed with anti-α tubulin as a loading control. See Supplementary Fig. 6E for full lane view of these data. (**e**) Southern blot analysis of DNA from three independent colonies of *cdc48-3*, *cdc48-3 ufd4Δ, ufd4Δ* cells and their respective (WT) isogenic controls were digested with XhoI and analysed by Southern blotting. (**f**) Proteins were prepared from α-factor-arrested WT, *cdc48-3*, *cdc48-3 ufd4Δ* and *ufd4Δ* cells grown at 25 °C (permissive temperature) and subjected to SDS–PAGE and western analysis of Est1 with mouse anti-Myc antibodies (Clontech) See Supplementary Fig. 6D for full lane view of these data.

Est1 is cell cycle regulated^19^ by a process that depends on both the E3 Ub ligase CDH1/APC^50^ and the proteasome^20^, although it is not clear whether either dependency is direct, and ubiquitinated Est1 (Est1-Ub) has not been detected. Our data suggest that when the Cdc48 complex is compromised, Est1 will be ubiquitinated but not degraded, leading to the accumulation of Est1-Ub. To test this model, we asked whether Est1-Ub is present in WT and *cdc48-3* cells by expressing Myc-tagged Est1 and His_6_-tagged ubiquitin under the control of the copper-inducible *CUP1* promoter in both WT and *cdc48-3* cells^51^. Lysates were prepared from log phase cells grown overnight in media containing copper and His_6_-ubiquitinated proteins were isolated using Ni-conjugated agarose beads (QIAGEN). Eluted proteins were separated by SDS–PAGE and analysed by western blots using anti-Myc (Clontech) to detect Est1 (Fig. 5a and Supplementary Fig. 6A) and anti-His antibodies (Novagen) to detect all ubiquitinated proteins (Supplementary Fig. 6B).

In both copper-treated strains, many proteins were present in the anti-His western blotting, but not in the non-copper-treated controls (Supplementary Fig. 6B, compare lanes 2 and 4 with 1 and 3). Myc-tagged Est1 was among the Ni-affinity isolated proteins from copper-treated cells, indicating that at least a fraction of the Est1 was ubiquitinated both in the presence and absence of the Cdc48 complex (Fig. 5a and Supplementary Fig. 6A). Although Est1-Ub was evident in both WT and *cdc48-3* cells, it was about 70–80 times more abundant in the latter (Fig. 5a and Supplementary Fig. 6A). After adjusting for Est1 being ∼40 times more abundant in *cdc48-3* versus WT cells, the fraction of Est1 that was ubiquitinated was almost twice as high in *cdc48-3* compared with WT cells. Although the Cdc48 complex is often described as recognizing polyubiquitinated proteins, in four independent experiments only mono-Ub Est1 was detected. Likewise, a recent paper reports that Cdc48 complexes in yeast and mammals extract monoubiquitinated transcription factors from DNA^52^.

To determine whether Est1 was ubiquitinated in a cell cycle-dependent manner, we arrested WT and *cdc48-3* cells in G1 phase with α-factor and G2/M phase with nocodazole, and isolated ubiquitinated proteins from both as in Fig. 5a. In both WT and *cdc48-3* cells, levels of mono-Ub Est1 were similar in G1 and G2/M phases (Fig. 5b and Supplementary Fig. 6C). Thus, the Cdc48 complex could extract Est1-Ub from telomerase in both G1 and G2/M phases.

### Cell cycle regulation of Est1 is lost in *cdc48-3* cells

Est1 is the only core telomerase subunit whose abundance varies with the cell cycle^1^,^8^,^19^. Est1 levels are approximately five times higher in G2/M (109±60.3 molecules per cell; here and elsewhere represents average±s.d.) than in G1 phase (20.3±11.6)^1^. To determine whether the Cdc48 complex is responsible for the cell cycle-regulated abundance of Est1, we arrested WT and *cdc48-3* cells in late G1 phase with α-factor. We then released cells from the arrest, allowing them to proceed through a synchronous cell cycle as monitored by fluorescence-activated cell sorting (FACS) (Fig. 5c). As shown previously, in WT cells Est1 was much less abundant in G1 phase than later in the cell cycle (Fig. 5d and Supplementary Fig. 6E). However, in *cdc48-3* cells Est1 levels were as high in G1 phase as they were throughout the rest of the cell cycle (Fig. 5d and Supplementary Fig. 6E). Thus, the low level of Est1 in G1 phase is Cdc48 dependent.

### Ufd4 affects telomere length and Est1 abundance

Budding yeast encodes ∼80 E3 Ub ligases^29^. Because three of these ligases Tom1, Ubr1 and Ufd4 were telomerase associated (Supplementary Data 1), we considered them as candidates for the E3 Ub ligase that modifies Est1. We also considered another E3 Ub ligase, Cdh1, which had been implicated previously in Est1 regulation^50^. We anticipated that deletion of the correct E3 Ub ligase would result in higher levels of Est1 and hence longer telomeres in otherwise WT cells. Est1 abundance in *cdc48-3* cells lacking the appropriate E3 ligase should be even higher than in *cdc48-3* cells alone but the short telomere phenotype of the strain should be suppressed. In two experiments, only *ufd4Δ* had all of the expected phenotypes (Fig. 5e,f and Supplementary Figs 6D and 7). Thus, Ufd4 is probably the E3 Ub ligase that cooperates with the Cdc48 complex to regulate Est1 abundance and degradation.

## Discussion

So far, MS analysis of telomerase has been accomplished only in Tetrahymena cells, as they undergo massive new telomere formation and immortalized human cells, both of which have atypically high levels of telomerase. As yeast telomerase is present in less than one complex per telomere, its isolation is challenging. We (and others) were not successful at purifying telomerase-associated proteins from budding yeast expressing endogenous levels of telomerase. Statistically robust preparations of telomerase-associated proteins required telomerase overexpression (Fig. 1d and Supplementary Fig. 1D) and optimized lysis conditions.

Telomerase overexpression had no detectable effects on growth rate or cell cycle progression (Fig. 1b). Moreover, even though telomeres were longer in the OE strains indicating that the OE telomerase was active, telomeres were not pathologically long as seen in certain mutant backgrounds, such as *cdc13-5* or *rap1*^*ts*^ cells^53^,^54^ (Fig. 1e). The robust association of all known telomerase subunits with the immunoprecipitated complex indicates that the isolated complex is authentic (Supplementary Data 1). Moreover, ∼30% of the telomerase-associated proteins were previously reported to affect telomeres, an enrichment over the ∼8% of yeast genes reported to affect telomere length (Supplementary Data 1).

The telomerase association of 35% of the 115 proteins, such as the Yku complex, was lost after DNase I treatment (Supplementary Data 1). The Ku complex is telomere associated in yeasts^55^ and mammals^56^. The DNase sensitivity of YKu70/80 and Sir4, another telomere structural protein, probably indicates that their association arises from telomerase that was telomere-associated at the time of cell lysis. However, we do not think these associations necessarily lack biological relevance, as Rap1, the most abundant telomere-binding protein, and the G-tail binding CST complex were not telomerase associated^57^. We speculate that telomerase-binding proteins such as the Yku complex have a particularly close proximity with telomerase when it is telomere bound, thus generating a favourable environment for interaction.

We identified telomerase-associated proteins from both G1 phase, when telomerase is not active, and G2/M phase, when it is. Proteins with roles in protein modification were more abundant in G1 than in G2/M phase telomerase. Degradation of telomerase subunits, such as Est1, or of other positive regulators of the enzyme in G1 phase could explain why telomerase is not active in G1 phase. In contrast, proteins involved in chromatin, silencing, transcription and translation were more highly represented in G2/M phase telomerase, perhaps reflecting a need to remodel telomeric chromatin for telomerase access. Nonetheless, all of the DNAse-resistant, telomerase-associated proteins were present with high specificity in both preparations, perhaps because factors that limit telomerase activity to late S/G2 phase are telomere rather than telomerase-associated. In addition, cell cycle regulation may be influenced in part by posttranslational modifications of telomerase subunits.

Our study identified an unanticipated pathway of telomerase regulation that involves the essential and multifunctional Cdc48-Npl4-Ufd1 complex and the E3 Ub ligase Ufd4. All four of these proteins were among the most significant telomerase-associated proteins in our MS analysis (Supplementary Data 1), yet none was linked previously to telomeres. The Cdc48 complex physically interacted with Est1 (Fig. 4d and Supplementary Fig. 4D) and affected its abundance and cell cycle regulation (Figs 4a and 5c,d, and Supplementary Figs 4 and 6E). When the abundance of the Cdc48 complex was impaired by temperature-sensitive mutations in any of its three subunits, Est1 levels increased as much as 40-fold (Fig. 4b and Supplementary Fig. 4B), at least a fraction of the accumulated Est1 was monoubiquitinated (Fig. 5a and Supplementary Fig. 6A), and its cell cycle-regulated abundance was lost (Fig. 5d and Supplementary Fig. 6E).

Overexpression of Est1 in WT cells results in telomere lengthening^49^ (see also Fig. 3d), consistent with its being a telomerase activator. However, hyper-elongation of telomeres on Est1 overexpression was Cdc48 dependent (Fig. 3d) and *cdc48-3* cells had short telomeres (Fig. 3a). These data suggest that Est1-Ub is a less effective telomerase activator than unmodified Est1. If this model is correct, deletion of the E3 Ub ligase responsible for Est1 ubiquitination should result in more Est1 and long telomeres. Indeed, deletion of the telomerase-associated E3 Ub ligase *UFD4* resulted in longer telomeres and more Est1 in WT cells (Fig. 5e,f and Supplementary Fig. 6D). Moreover, deleting *UFD4* from *cdc48-3* cells increased Est1 abundance even more but had a positive effect on telomere length, presumably because the fraction of ubiquitinated Est1 was reduced and therefore Est1 was better able to promote telomerase activity.

**Figure 6:**
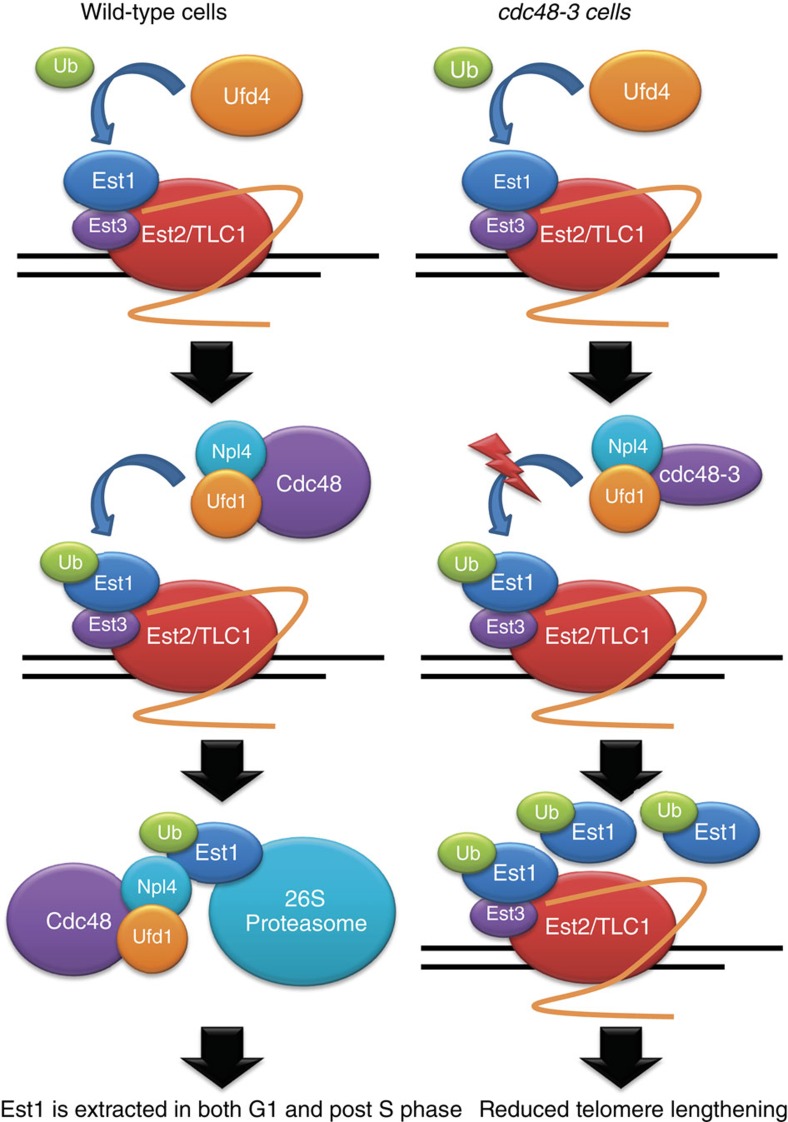
Model for Cdc48-Npl4-Ufd1 regulation of telomerase activity and telomere length. The Cdc48 complex delivers proteins to the proteasome for degradation and Est1 abundance is cell cycle regulated by a proteasome-dependent process. We show that the Cdc48 complex is telomerase associated. We propose that Est1 is ubiquitinated by Ufd4 and this modified form is less capable than unmodified Est1 at fulfilling the activation function of Est1. The Ub binding subunits of the Cdc48 complex recognize and bind Est1-Ub, while Cdc48 catalyses its removal from telomerase, thereby rendering telomerase inactive. We propose that removal can occur in G1 phase to prevent premature assembly of telomerase or in G2/M phase to promote telomerase disassembly. The Cd48 complex brings Est1-Ub to the 26S proteasome, thereby reducing its abundance.

Why is it important to limit holoenzyme assembly to a small window in the cell cycle? A cell cycle-regulated holoenzyme is probably not important for telomere length control, because when the holoenzyme is assembled prematurely in G1 phase, it does not lengthen telomeres^20^. However, double-strand breaks can occur anytime in the cell cycle and their repair by telomere addition, which results in loss of all sequences distal to the site of the break, is a much less favourable outcome than repair by homologous or even non-homologous recombination. In the absence of the Cdc48 complex, Est1 was more abundant and no longer cell cycle regulated (Fig. 5c,d and Supplementary Fig. 6E). Based on these data, we propose that the Cdc48 complex is part of a regulatory circuit that prevents premature assembly of the telomerase holoenzyme in G1 and early S phase (Fig. 6). However, the Cdc48 complex was also abundantly associated with telomerase in G2/M phase and Est1 was ubiquitinated to similar extents in G1 and G2/M phase (Fig. 5b and Supplementary Fig. 6C), suggesting that the complex may also function to disassemble the holoenzyme at the end of the cell cycle (Supplementary Data 1). Such an activity would be similar to the role of Cdc48-Npl4-Ufd1 in disassembling the replisome at the end of S phase^58^,^59^. Moreover, in genetic backgrounds that allow the precocious assembly of the replisome in G1 phase, the Cdc48 complex can also disassemble it at this time^58^. Thus, there is precedent for proposing that the Cdc48 complex can prevent telomerase holoenzyme formation in G1 phase and promote its disassembly at the end of the cell cycle.

In carrying out its diverse functions, the Cdc48 complex acts on its substrates when they are bound to a subcellular structure, such as the endoplasmic reticulum or chromatin^58^,^59^. This property in combination with our model provides an explanation for an otherwise puzzling aspect of telomere biology, the telomere bound but unengaged association of the catalytic core of telomerase, Est2 and TLC1, in G1 phase when telomerase is not active^19^. We speculate that the transient chromatin association of a precociously assembled telomerase holoenzyme is required to make it a substrate for Cdc48-mediated disassembly.

In summary, we propose that the Cdc48 complex prevents premature assembly of the telomerase holoenzyme early in the cell cycle and promotes its regulated disassembly at the end of the cell cycle. These actions would narrow the cell cycle window in which *de novo* telomere addition can occur. Given the conserved functions of the Cdc48 complex in DNA repair and replication, its role in telomerase regulation may also be conserved.

## Methods

### *S. cerevisiae* growth conditions and strains

All experiments were performed in the W303 background (*RAD5 leu2-3,112 trp1-1 can1-100 ura3-1 ade2-1 his3-11,15*). Genotypes of the yeast strains used in this study are listed in Supplementary Table 1. Telomerase was prepared for MS analyses from a strain in which Est1, Est2, Est3 and Tlc1 were OE from the *GAL1* promoter at their endogenous loci and as the only copies of the genes in the cell. Telomerase OE strains were cultured in 2% galactose, because they senesce on glucose medium. In addition, Est1 and Est2 were expressed as GFP fusion proteins: the amino-terminal GFP tag was separated from *EST1* or *2* by a five-glycine linker, which increases the functionality of epitope-tagged *S. cerevisiae* proteins^60^. The gene encoding the Bar1 protease was deleted by insertion of the NatMX cassette^61^. A *bar1*::NatMX strain expressing GFP fused to a nuclear localization signal (*NLS*) from SV40 T antigen at the *URA3* locus (*ura3*::*GAL-GFP-NLS*) and expressed from the *GAL1* promoter was used in parallel experiments to assess nonspecific interactions with the GFP tag.

Cell cycle arrest and FACS analysis were carried out^19^. Briefly, the cells were cultured in YEP-rich media to OD_660_ ∼0.15 and transferred to media containing 20 ng ml^−1^ α-factor (Sigma) or 15 μg ml^−1^ nocodazole (Sigma-Aldrich) for 4 h at 30 °C (or for 6 h at 25 °C for temperature-sensitive strains) for arrest at G1 (α-factor) or G2/M phase (nocodazole). Cell cycle position was verified by FACS analyses.

### Immuno-affinity purification of telomerase

For MS experiments, two litres of cells were grown to mid-log phase (OD_660_=0.5) in YEP+galactose and were harvested by centrifugation at 4 °C for 10 min at 6,118*g*. Cell pellets were resuspended in freezing buffer (20 mM Na-HEPES, 1.2% polyvinylpyrrolidone (W/V), pH 7.4) containing 1 × protease inhibitor cocktail (0.02 mg ml^−1^ pancreas extract, 0.05 mg ml^−1^ pronase, 0.005 mg ml^−1^ thermolysin, 0.0015, mg ml^−1^ chymotrypsin, 0.33 mg ml^−1^ papain; Roche) and were frozen dropwise in liquid nitrogen^62^. The frozen cell pellets were cryogenically ground using a Retsch MM301 Mixer Mill (15 cycles × 2.5 min at 30 Hz; Retsch, Newton, PA), to achieve a minimum of 85% cell lysis, as assessed using light microscopy. Approximately 12 g of frozen cell powder were resuspended in lysis buffer (100 mM Hepes KOH, pH 7.9, 300 mM potassium acetate, 10 mM magnesium acetate, 10% glycerol, 0.1% NP-40, 2 mM EDTA, 2 mM β-glycerophosphate, 50 mM NaF, 1 mM dithiothreitol, 1 × protease inhibitor cocktail (Roche))^63^ in a ratio of 5 ml of lysis buffer per 1 g of cells. Cell lysates were homogenized using a PT 10–35 Polytron (Kinematica) for three cycles of 10 s each, with a 1-min ice incubation between each set. Insoluble material was removed by centrifugation at 4 °C for 10 min at 10,876*g*. The supernatant was incubated for 30 min at 4 °C with ∼20 mg of M-270 epoxy magnetic beads (Life Technologies) conjugated with 50 μg of in-house-purified polyclonal rabbit anti-GFP antibodies^44^,^64^. Following incubation, the beads were separated from the solution on a magnet and were washed six times with 1 ml cold lysis buffer. Proteins were eluted from the beads in 40 μl of 1 × LDS sample buffer (Life Technologies) and incubated with agitation for 10 min at room temperature, followed by incubation for 10 min at 70 °C. Eluted proteins were alkylated with 50 mM iodoacetamide for 30 min at room temperature in the dark.

The isolated protein complexes were separated on a 4%–12% bis-Tris NuPAGE precast gradient gel (Life Technologies), to reduce sample complexity, and stained with SimpleBlue Coomassie (Life Technologies) for visualization of proteins. Samples were prepared for in-gel digestion by excising gel bands as 1-mm sections. Gel sections from a single immuno-isolation were pooled into approximately ten equal fractions and placed into individual wells of a 96-well plate. The gel pieces were destained in 50 mM ammonium bicarbonate (ABC), 50% acetonitrile (ACN) at room temperature with gentle shaking for 15 min. Next, the samples underwent two rounds of dehydration in 100% ACN and rehydration in 50 mM ABC. After a final, third dehydration step in 100% ACN, the gel pieces were resuspended in a solution of 50 mM ABC and 12.5 ng μl^−1^ sequencing grade modified trypsin (Promega) for overnight digestion at 37 °C. Peptides were extracted from the gel pieces in 0.5% formic acid (FA) at room temperature with gentle shaking for 4 h, followed by a second extraction in 0.5% FA/50% ACN at room temperature with gentle shaking for 2 h. The peptides were concentrated by vacuum centrifugation and acidified with 1% FA before a final volume of 9 μl for MS analysis.

To determine whether the observed protein interactions were or were not dependent on an association with DNA, the experiments were repeated by comparing cells treated (10 μg ml^−1^ for 30 min) or not treated with DNase I (Sigma-Aldrich). The experimental workflow was the same as above, except the starting material for each isolation was 1 litre of cells. This comparison allowed us to determine which interactions were likely mediated by association with DNA. In some cases, proteins that passed our specificity criteria (described below) in the initial large-scale experiment using 2 l of cells were not identified in the smaller scale experiment (1 litre cells). These experiments are labelled as ‘DNA-dependency inconclusive' (Fig. 2a and Supplementary Data 1).

### Mass spectrometry

Before direct infusion, samples were desalted using StageTips and an Empore C18 filter (3M)^65^ and MS analyses were performed^66^. Briefly, tryptic peptides were separated and analysed by nanoscale liquid chromatography electrospray ionization tandem MS (MS/MS) on a Dionex Ultimate 3000 RSLC system (Dionex Corporation) (running mobile phases: A (aqueous), 0.1% FA/H_2_O; B (organic), 0.1% FA/97% ACN/2.9%H_2_O) directly coupled to an LTQ-Orbitrap XL (ThermoFisher Scientific) instrument. Samples were separated on a nanocapillary reverse-phase PepMapC18 analytical column (75 μm by 15 cm; particle size 1.8 μm) (Dionex Corporation) with a 90-min linear gradient (4%–35% mobile phase B). Mass spectra were acquired over the *m*/*z* range from 350 to 1,700 (*r*=30,000 at *m*/*z* 400), with data-dependent selection and fragmentation of the top ten most intense precursor ions by collision-induced dissociation. Precursor ions were selected in the ion trap (isolation width=2.00 Da, normalized collision energy=30%, activation *q*=0.250, activation time=30 ms). MS/MS data searches were carried out using the following parameters: precursor mass tolerance=0.02 Da; fragment mass tolerance=0.5 Da.

### Data and network analyses

MS/MS spectra from raw files corresponding to single biological samples were extracted using Proteome Discoverer (v.1.3, ThermoFisher Scientific) running the SEQUEST (version 1.20) search engine against a database of *S. cerevisiae* protein sequences (UniProt-SwissProt, 2010-11) and common contaminant sequences. Spectra were searched against indexed peptide databases generated from both the forward sequences and the reversed, concatenated protein sequences. The search parameters included full enzyme specificity with a maximum of two missed cleavages. Parent and fragment ion mass tolerances were limited to 10 p.p.m. and 0.5 Da, respectively. All searches incorporated static modification of cysteine by carbamidomethylation (+57 Da) and variable modifications of methionine by oxidation (+16 Da) of serine, threonine and tyrosine by phosphorylation (+80 Da), and of lysine by acetylation (+42 Da). Percolator in ProteomeDiscoverer and PeptideProphet in Scaffold (v 3.3.1; Proteome Software) were used to calculate probabilities for spectral matches^67^. The X!Tandem (GPM 2010.12.1.1, Beavis Informatics) subset database refinement search was selected to include additional variable modifications: deamidation of asparagine and glutamine (+1 Da), pyro-Glu formation of peptide N-terminal glutamate (+17 Da) and diglycine modification of lysine (+114.1 Da), with the high mass accuracy option enabled.

Scaffold confidence filters were selected to reduce peptide false discovery rates (<1%). Unweighted spectrum counts from resulting protein lists were exported and used for subsequent analyses. To determine specificity of interaction, proteins were filtered using the SAINT algorithm. A SAINT specificity filter of 0.8 was selected to retain high confidence interactions. Proteins passing the SAINT *P*>0.8 in either α-factor-treated or nocodazole-treated isolations were included in the merged set of proteins for fold-change analyses. Unweighted spectrum counts for individual proteins were normalized to GFP spectrum counts from each isolation and averaged. Fold changes are represented as the log_2_-transformed ratios of average spectrum count values (G_1_/G_2_M). Protein networks were generated using STRING-db^40^ and Cytoscape^41^, incorporating the built-in Gene Ontology annotation functionality for clustering of protein complexes (Gene Ontology Full: geneontology.org/ontlogy/gene_ontology_edit.obo; Gene Association File for *S. cerevisiae*: geneontology.org/gene-associations/gene_association.sgd.gz, curator: SGD).

Gene Ontology enrichment analyses were performed using the Gene Functional Annotation Tool operating on the DAVID Bioinformatics 6.7 online platform (NIAID/NIH)^44^,^68^. Proteins identified in telomerase isolations were uploaded as a single protein list using SGD_ID annotations. Proteins enriched in either G1 isolations were uploaded as a separate protein list. Corresponding GO annotations were searched using the whole *S. cerevisiae* genome as background library. Functional Annotation Clustering was performed using the following parameters: Gene Ontology=GOTERM_BP_FULL, Classification Stringency=Highest). DAVID cluster enrichment scores are expressed as the geometric mean of *P*-values for individual genes within each cluster.

### Western blotting

Cells were grown in rich medium to mid-log phase for asynchronous cell growth or to early log phase for cell cycle synchrony. Cells were pelleted, resuspended in 0.1 M NaOH and pelleted. The whole-cell protein was extracted using protein sample buffer^69^. Protein were separated in 8% SDS–PAGE gels and transferred to nitrocellulose membrane (Millipore). Membranes were blocked in TBST (10 mM Tris, 150 mM NaCl, 0.05% Tween-20) with 5% milk. Probes used for western blot analysis included mouse anti-GFP monoclonal serum (Roche, 11814460001, dilution 1:1,000), mouse anti-Myc monoclonal antibodies (Clontech, 631206, dilution 1:500), horseradish peroxidase-conjugated goat anti-rabbit (Bio-Rad, 1706515, dilution 1:5,000), goat anti-mouse immuno-globulin G polyclonal antibodies (Bio-Rad, 1721011, 1:3,000) and rabbit anti-goat immunoglobulin G polyclonal antibodies (Abcam, 6,741, 1:5,000). The full lane view of the these data are shown in the Supplementary Figs.

### Co-immunoprecipitation of Cdc48

Cells were grown asynchronously in rich medium to mid-log phase. Extracts were prepared by the glass-bead lysis in TMG-50 (10 mM Tris-HCl, pH 8.0, 1 mM MgCl2, 10% (v/v) glycerol, 50 mM NaCl, 0.1 mM dithiothreitol, 0.1 mM EDTA, Complete Mini EDTA-free protease inhibitor (Roche)). Total protein was adjusted to 0.5% (v/v) Tween-20 and 200 U ml^−1^ of RNasin (Promega) and SuperRNasin (Ambion) and incubated overnight with goat polyclonal anti-Cdc48 antibodies (Abgent) at 4 °C. Dynabeads Protein G (Life Technologies) equilibrated with TMG-50 with 0.5% (v/v) Tween-20 were added and incubated for 4 h at 4 °C. Beads were washed three times with TMG-50 with 0.5% (v/v) Tween-20, and once with TMG-50. The co-immunoprecipitated samples were resuspended in TMG-50 and protein was extracted using protein sample buffer.

### Southern blotting

Genomic DNA was isolated from cells using a MasterPure Yeast DNA Isolation kit (Epicentre Biotechnologies), genomic DNA was digested with XhoI, separated on 0.8% agarose gels, transferred to Hybond N+ nylon membrane (GE Healthcare) and hybridized with a randomly primed ^32^P-labelled telomere probe isolated from pCT300 (ref. 70).

### Telomere position effect

TPE was assessed in strains having *URA3* adjacent to the left telomere of chromosome VII (ref. 47). Cells growing in YEP+2% glucose (or 2% galactose for OE strains) were diluted to a concentration of 2 × 10^7^ cells per ml. Then, tenfold serial dilutions of the cell suspensions were made and 3 μl of each dilution was spotted onto synthetic complete (YC) plates, YC plates without uracil (YC-ura) and YC plates containing 0.1% 5-FOA (Toronto Research Chemical) (YC+5-FOA). The plates were incubated at 25 °C for 3 days.

### Purification of ubiquitinated proteins

Cells were transformed with a plasmid (YEp352-6HisUb) harbouring 6His-tagged ubiquitin under the *CUP1*-inducible promoter^51^. The cells were grown in media lacking uracil (to select for the plasmid). 6His-Ubiquitin was induced by 24 h treatment with 0.1 mM CuSO_4_. Cells were harvested and lysed with guanidinium lysis buffer (6 M guanidine hydrochloride, 100 mM sodium phosphate buffer pH8.0, 10 mM Tris-HCl pH8.0, 10 mM imidazole, 10 mM β-mercaptoethanol, 0.1% Triton X-100, 2.5 mg ml^−1^ N-methyl maleimide, 0.1 mM MG-132, 1 × protease inhibitor). Purification of 6His-ubiquitinated proteins was performed using the Ni-NTA (Ni^2+^-nitrilotriacetic acid) agarose beads (QIAGEN). The beads were washed with urea buffer (8 M urea, 100 mM sodium phosphate buffer pH 6.4, Tris-HCl pH 6.4, 10 mM imidazole, 10 mM β-mercaptoethanol, 0.1% Triton X-100) and subsequently eluted with protein sample buffer. Eluted protein samples were separated by SDS–PAGE and analysed by western blots using anti-Myc (Clontech) and anti-His antibodies (Novagen).

## Additional information

**How to cite this article:** Lin, K.-W. *et al*. Proteomics of yeast telomerase identified Cdc48-Npl4-Ufd1 and Ufd4 as regulators of Est1 and telomere length. *Nat. Commun.* 6:8290 doi: 10.1038/ncomms9290 (2015).

## Supplementary Material

Supplementary Information Supplementary Figures 1-7, Supplementary Table 1 and Supplementary References.

Supplementary Data 1 Proteins co-immunoprecipitated with telomerase in alpha-factor treatment (G1 phase) or nocodazole treatment (G2/M phase). Bold and Italic represent proteins resistant to DNase I treatment; Dark red represent DNA-dependency inconclusive; X Known telomerase component; # Proteins known to affect telomere length from genome wide screen; * Proteins associated with telomere biology from another criterion.

## Figures and Tables

**Table 1:** Telomerase-specific protein interactions associate dynamically with telomerase during cell cycle progression.

**EST1/EST2 interactions**	**G1 vs G2/M**	**SAINT scores**	
		**Synch.**	
**Gene**	**Log2 ratio**	**G1**	**G2/M**
*Telomerase*			
EST1	−0.04	1.00	1.00
EST2	0.10	1.00	1.00
EST3	0.65	1.00	1.00
			
*Cdc48-Npl4-Ufd1*			
CDC48	0.50	1.00	1.00
NPL4	0.71	1.00	1.00
UFD1	0.20	1.00	1.00
			
*Sm complex*			
SMX3	0.90	1.00	0.94
SMB1	−0.32	1.00	1.00
SMD1	0.46	1.00	1.00
SMD3	0.26	1.00	1.00
SMD2	0.42	1.00	0.97
SME1	0.41	1.00	0.67
			
*Ku70/Ku80*			
YKU70	0.61	1.00	1.00
YKU80	0.46	1.00	1.00
			
*RNase P*			
POP1	0.14	1.00	1.00
POP6	0.20	1.00	1.00
POP7	−0.06	0.99	0.99
			
*Ubiquitinylation*			
APC1	2.02	1.00	0.24
TOM1	0.44	1.00	1.00
UBP15	1.15	1.00	0.80
UBR1	0.27	1.00	1.00
UBX2	0.51	1.00	1.00
UFD4	0.37	1.00	1.00
RPT4	0.78	1.00	0.97
SHP1	0.34	1.00	1.00
STI1	−0.32	1.00	1.00
			
*Proteasome*			
ECM29	1.05	0.98	0.87
PRE10	0.17	0.99	0.97
PRE2	1.08	0.87	0.65
PRE3	0.80	1.00	0.94
PRE5	0.28	1.00	1.00
PRE6	0.73	0.96	0.94
PRE7	0.14	0.89	0.93
PRE8	0.31	0.85	0.96
RPN11	−1.62	0.93	0.34
RPN13	0.87	0.96	0.56
RPN1	0.48	1.00	1.00
RPN2	0.64	1.00	1.00
RPN3	0.49	0.93	0.88
RPN5	0.61	1.00	0.99
RPN6	0.70	0.99	0.90
RPN7	0.91	0.93	0.76
RPN9	0.63	0.98	0.79
RPT1	0.20	1.00	1.00
RPT2	0.34	0.99	0.98
RPT3	1.00	1.00	0.95
RPT5	1.21	1.00	0.98
SCL1	0.74	0.99	0.97
			
*DNA repair/chromatin organization*			
CKB2	−0.08	0.80	0.05
ESC1	0.22	0.48	0.84
GCN2	0.04	0.10	0.97
MEC3	−0.70	0.47	0.92
MLP1	−0.79	0.00	0.80
NAP1	0.72	1.00	1.00
PAA1	−0.37	1.00	1.00
PTC3	−0.18	0.95	0.99
RAD50	0.81	0.70	0.97
SIR4	0.20	0.94	0.00
SWC4	1.79	0.91	0.03
TIF1	−0.37	0.81	0.56
YMR247C	0.21	1.00	0.99

Proteins co-isolated with telomerase during G1 and G2/M are grouped into functional categories based on gene ontologies and STRING connectivity. Changes in the abundance of protein associations are indicated as the log-transformed ratios of normalized spectrum counts (log2 ratio) for individual cell cycle stages. Interaction specificity for individual proteins was determined using the SAINT algorithm with a specificity score threshold of SAINT *P*>0.80 for either G1 or G2/M isolations.
