# Supplementary material for: Proteomics of yeast telomerase identified Cdc48-Npl4-Ufd1 and Ufd4 as regulators of Est1 and telomere length
Source: Nat Commun. 2015 Sep 14;6:8290. doi: 10.1038/ncomms9290 (PMC4579843; doi:10.1038/ncomms9290)
Supplement: Supplementary Information — Supplementary Figures 1-7, Supplementary Table 1 and Supplementary References. [file ncomms9290-s1.pdf]

## Supplementary Figures

**Supplementary Figure 1.**

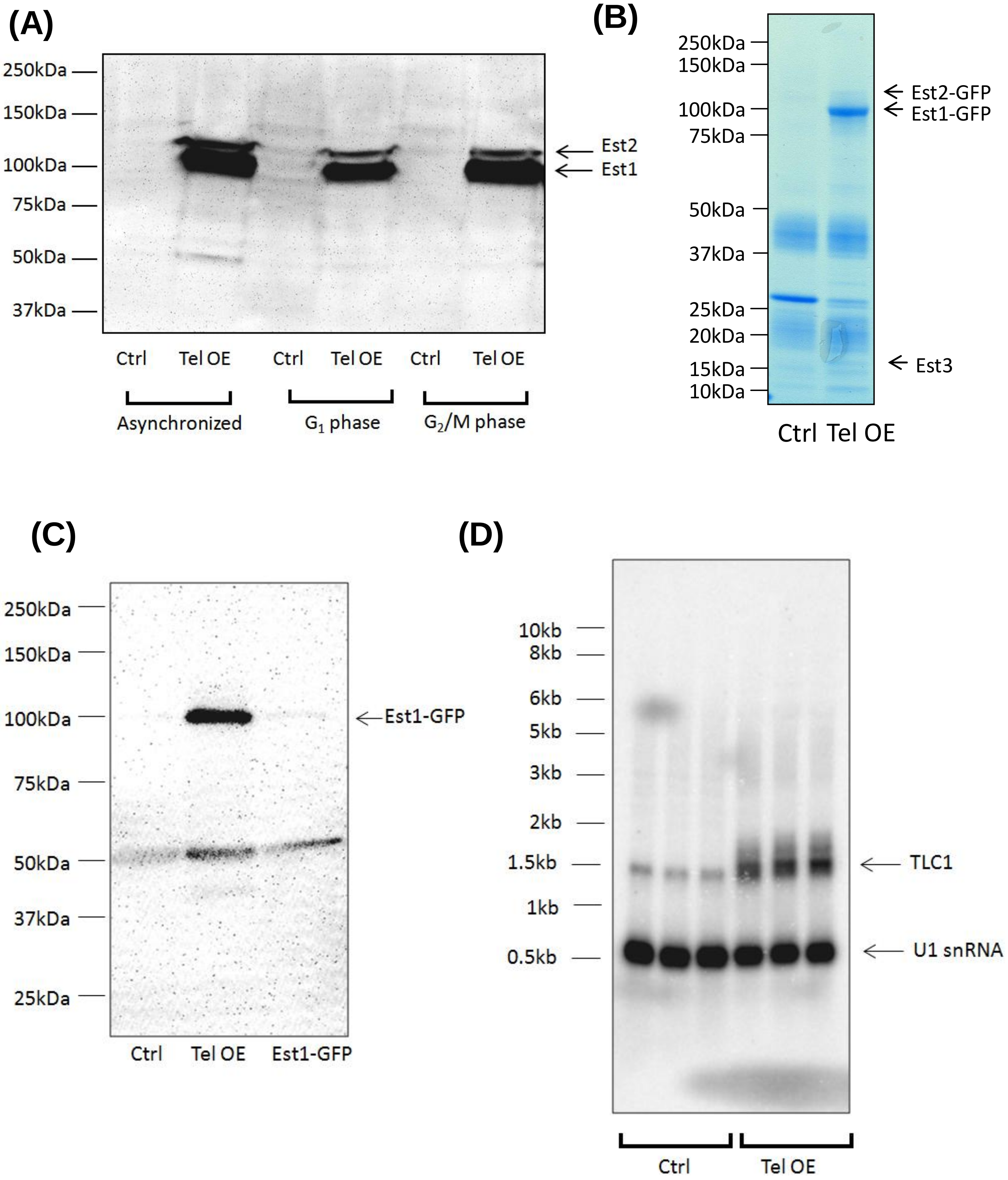

**Supplementary Figure 1.** (A) Western analysis of telomerase over-expressing cells using an anti-GFP antibody confirmed high expression of Est1 and Est2 in telomerase over-expressing cells (Tel OE). In asynchronous cells, Est1 is about twice as abundant as Est2. Although this western suggests that the Est1:Est2 ratio was much higher than this in telomerase over-expressing cells, the spectral counts of the two proteins indicate an Est1:Est2 ration of 2:1. The higher apparent Est1: Est2 ratio in the western is probably due to degradation of Est2, which generates a partial Est2-GFP polypeptide that migrates with Est1-GFP. (B) Coomassie-stained of the protein extracts immunoprecipitated with anti-GFP antibodies. The control is proteins associated with nuclear localized GFP and Tel OE is from the strain in which the four core telomerase subunits are overexpressed. The positions of Est2-GFP, Est1-GFP, and Est3 in OE strain are indicated (Tel OE), although Est3 expression in the Coomassie stain is not prominent. (C) Full lane view of Figure 1C. (D) Full lane view of Figure 1D.

**Supplementary Figure 2.**

**I**

**(A)**

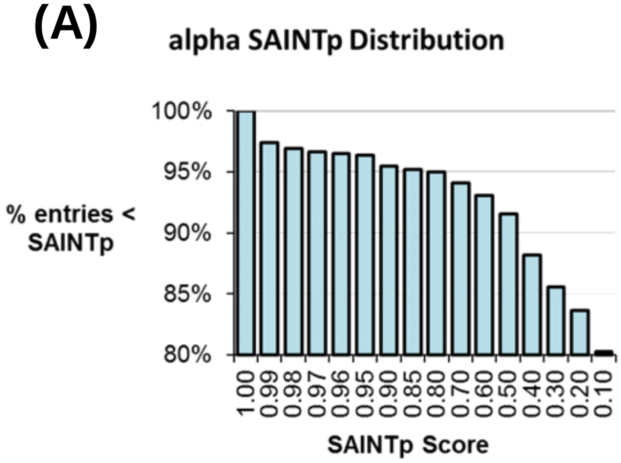


**(B)**

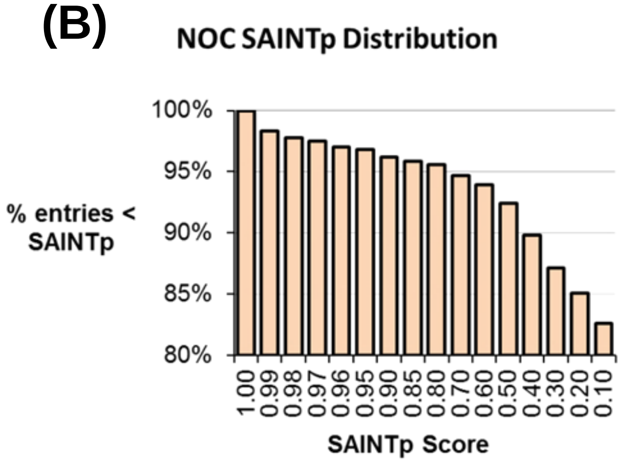


**II**

**(A)**

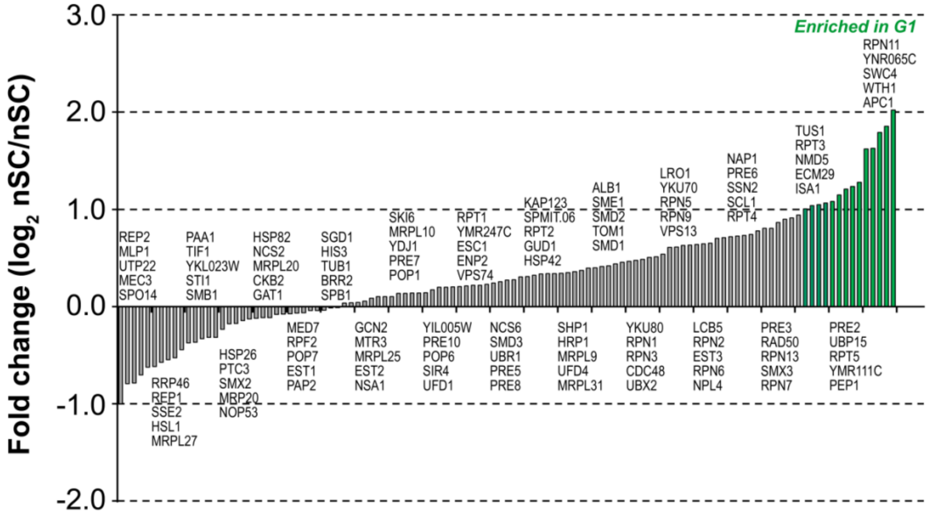


**(B)**

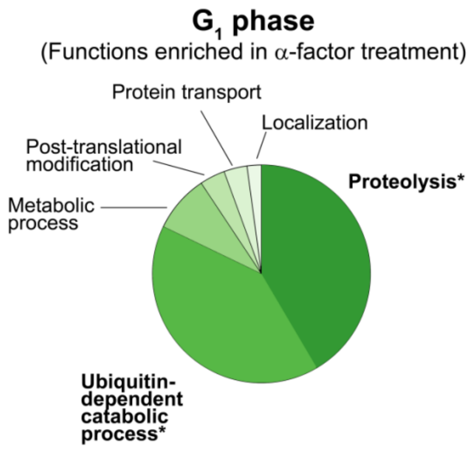

**Supplementary Figure 2.** I. Binned distributions of SAINT scores determined for proteins isolated in (A) G1 (alpha) isolation or (B) G2/M (NOC). SAINT comparisons were performed using EST1/2 bait isolations (n=2) and control GFP isolations (n=2). II. (A) Fold-enrichment in telomerase-specific protein associations of inactive (G1) and active (G2/M) telomerase. Fold enrichment scores are calculated as the $\log_2$-transformed ratios of spectrum counts (nSC/nSC) per protein after normalization to GFP abundance. Proteins enriched ($\log_2$ (nSC/nSC)>1) in interaction abundance during G1 are shown in green. (B) Functional enrichment analysis of interactions enriched in G1 isolations. Comparisons were performed against a whole *S. cerevisiae* genome background gene list, wedge sizes represent relative DAVID group enrichment scores (min = 0.24/max = 4.76), * represent significantly enriched functions ($p<0.05$, modified Fisher Exact test (EASE)).

**Supplementary Figure 3.**

**I**

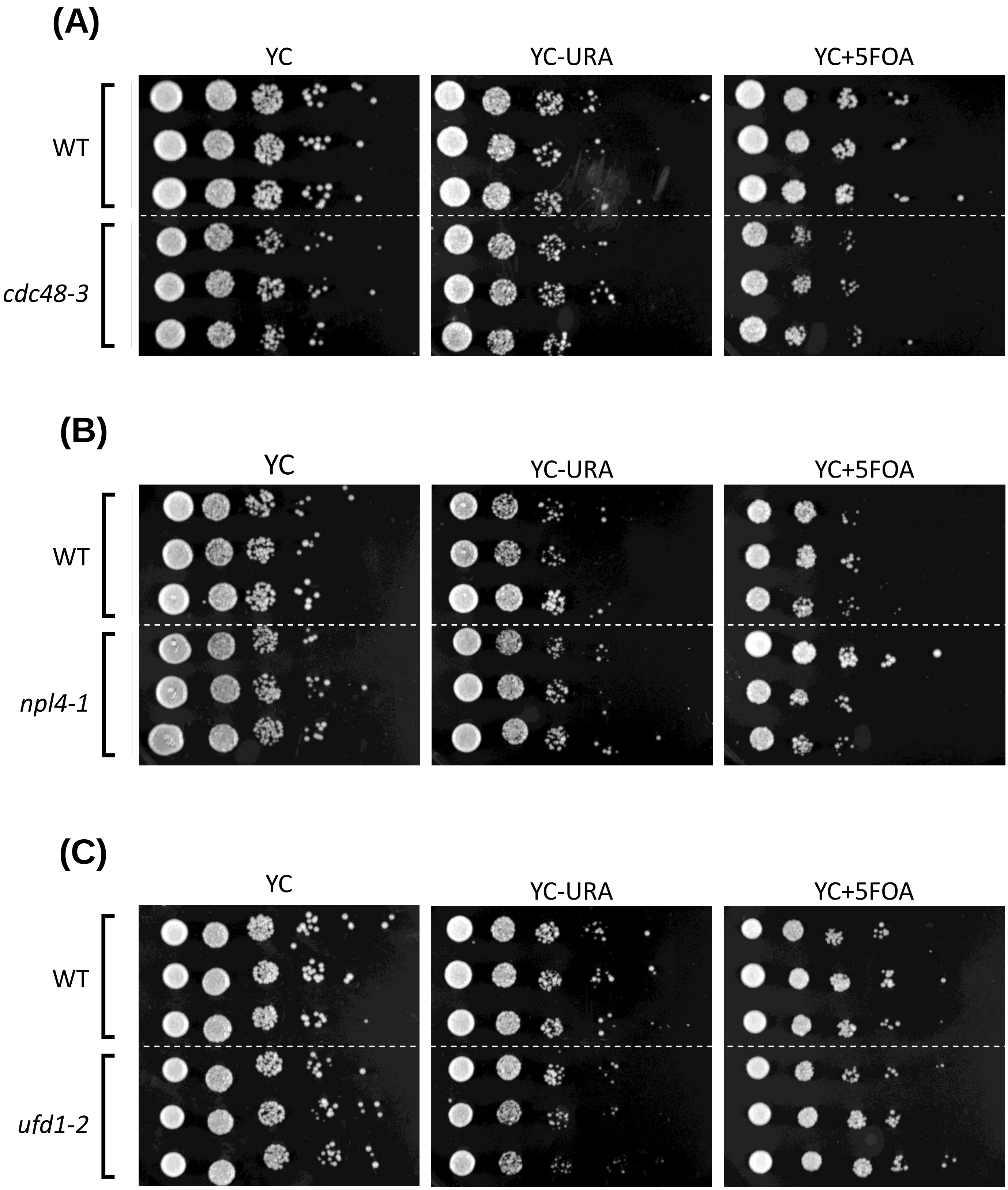

II

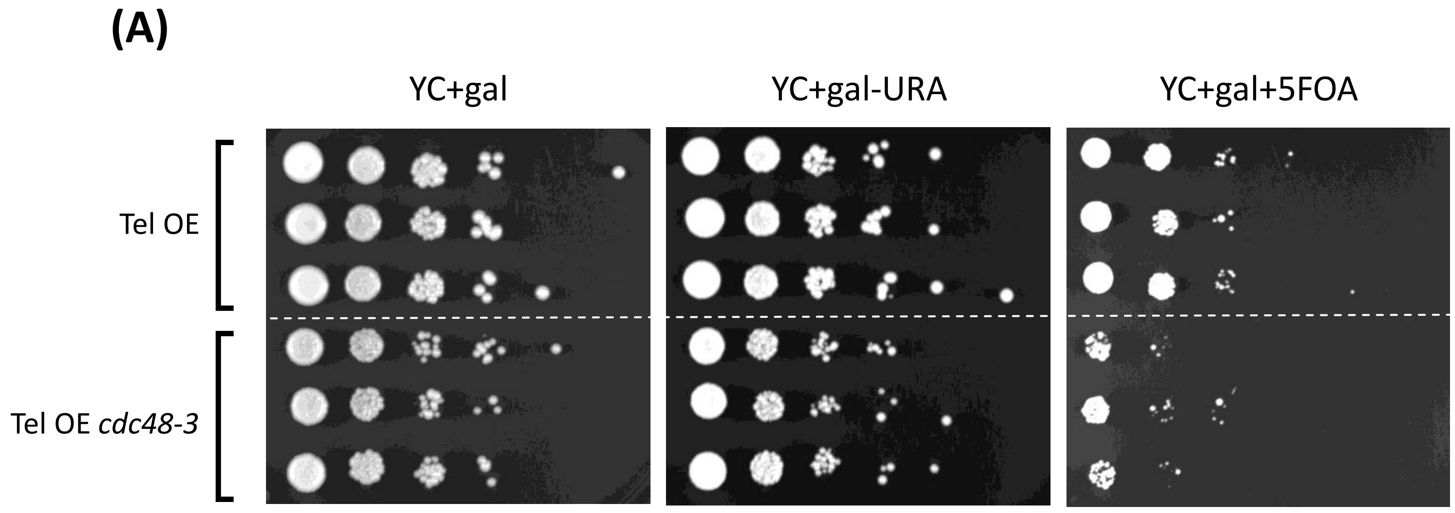

(A)
YC+gal
YC+gal-URA
YC+gal+5FOA
Tel OE
Tel OE *cdc48-3*


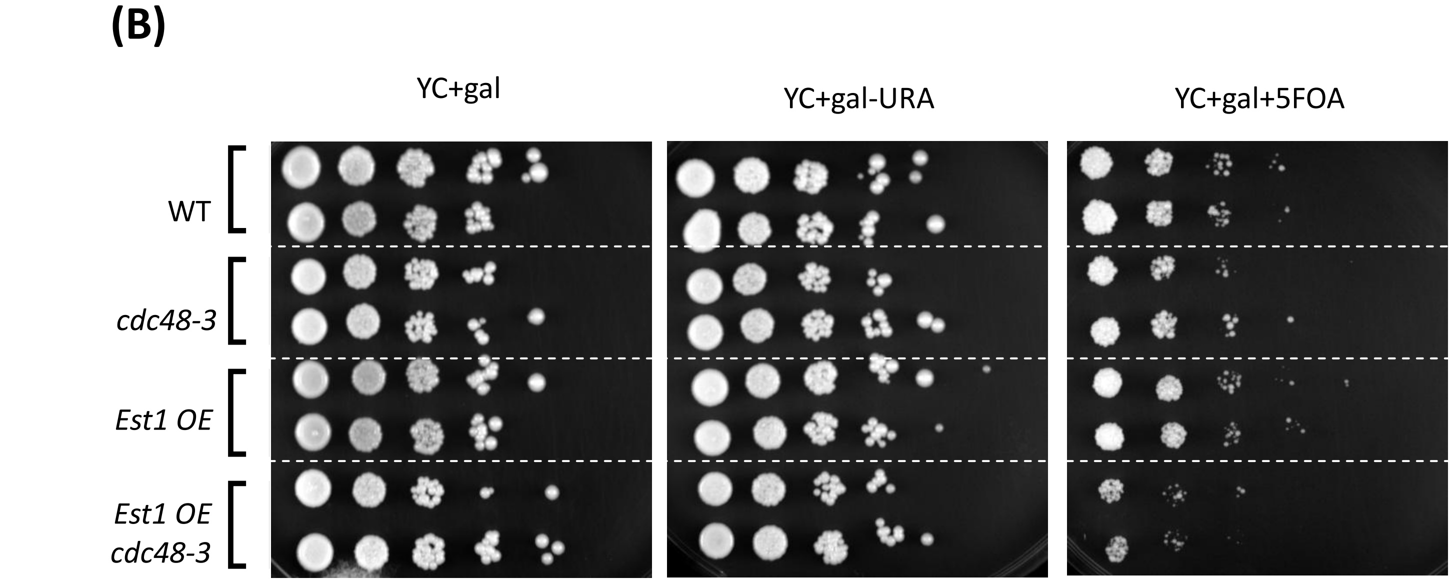

(B)
YC+gal
YC+gal-URA
YC+gal+5FOA
WT
*cdc48-3*
*Est1 OE*
*Est1 OE cdc48-3*

**Supplementary Figure 3.** Telomere position effect is modestly reduced in *cdc48-3* cells (A) Cells from *cdc48-3* or the isogenic WT strain, both with *URA3* positioned next to the left telomere of chromosome VII, were spotted on rich medium plates (YC, Y complete), rich medium lacking uracil (YC- URA), or rich medium containing uracil and 5-fluoro-orotic acid (5-FOA). Each successive spot is a ten-fold dilution of the spot to its left. Cells were grown at 25°C. Only cells that do not express the telomere adjacent *URA3* gene grow on FOA. (B) *npl4-1* and (C) *ufd1-2* cells with their isogenic WT controls were treated as in panel A. II. Telomere position effect in *cdc48-3* telomerase and Est1 over-expressing cells. Experiment is the same as in I except that cells are plated on medium containing galactose and in panel B, WT and *cdc48-3* cells over-express Est1.

**Supplementary Figure 4.**

**(A)**

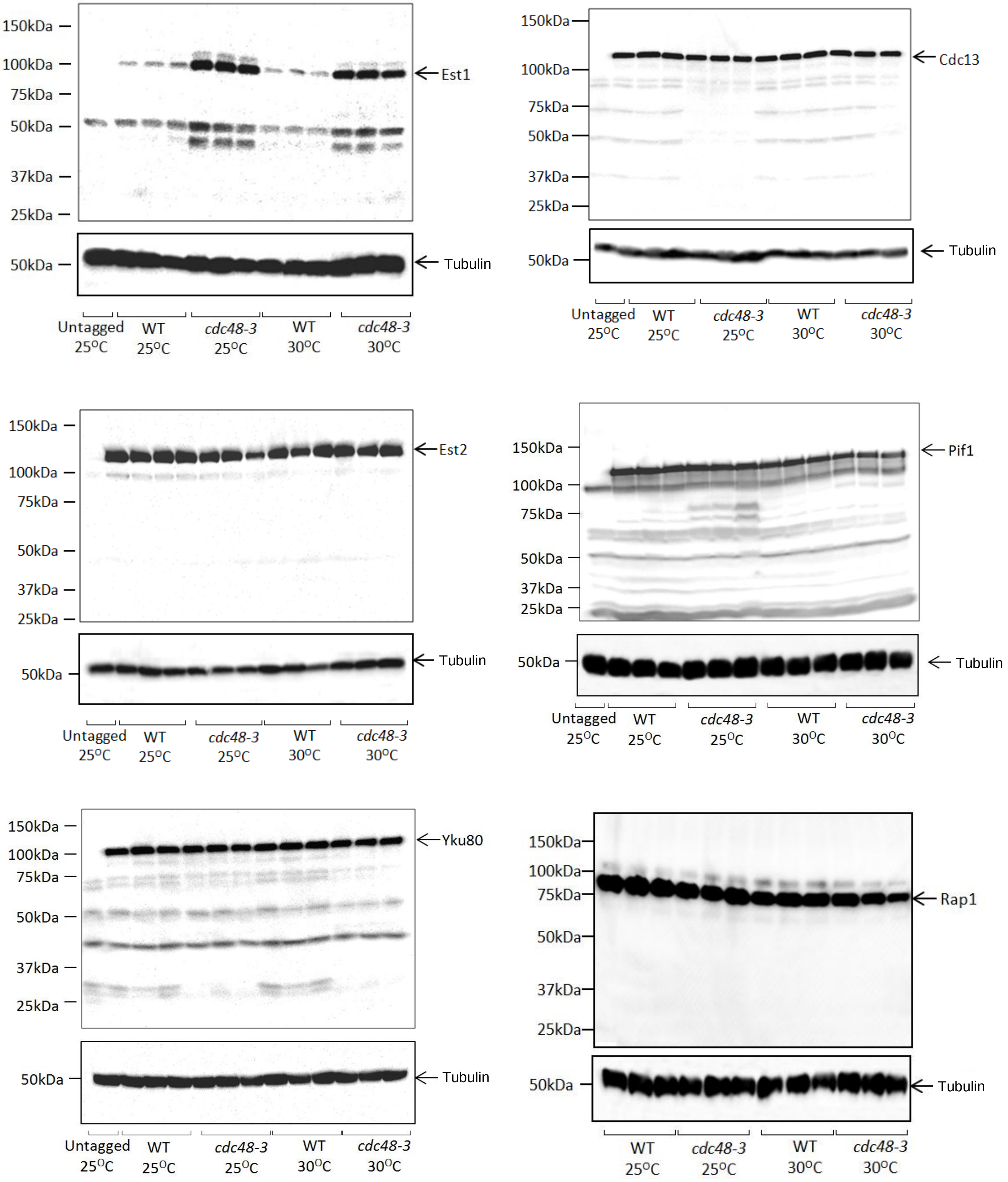

(B)

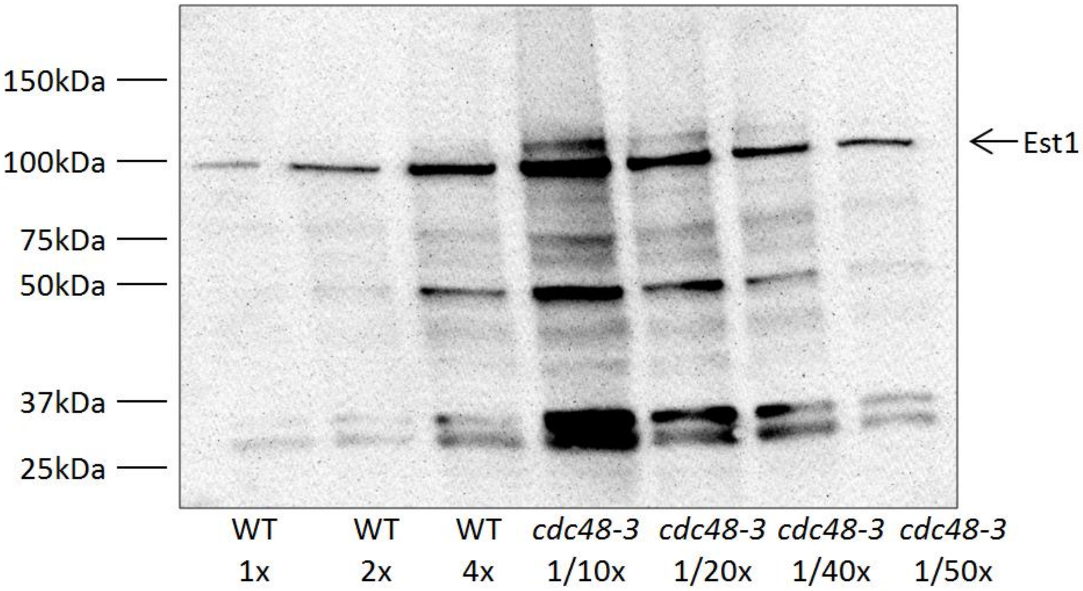
150kDa
100kDa
75kDa
50kDa
37kDa
25kDa
Est1
WT 1x
WT 2x
WT 4x
cdc48-3 1/10x
cdc48-3 1/20x
cdc48-3 1/40x
cdc48-3 1/50x

(C)

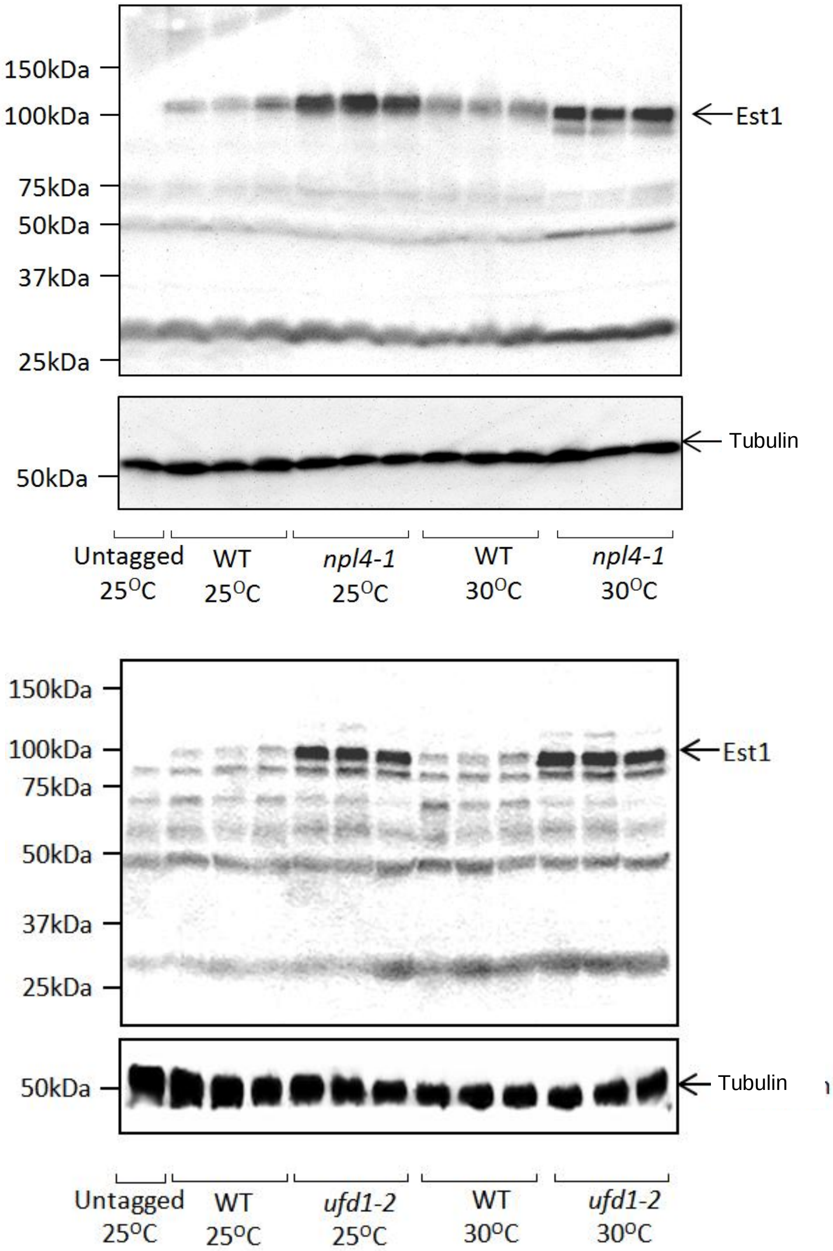
150kDa
100kDa
75kDa
50kDa
37kDa
25kDa
Est1
50kDa
Tubulin
Untagged 25°C
WT 25°C
npl4-1 25°C
WT 30°C
npl4-1 30°C
150kDa
100kDa
75kDa
50kDa
37kDa
25kDa
Est1
50kDa
Tubulin
Untagged 25°C
WT 25°C
ufd1-2 25°C
WT 30°C
ufd1-2 30°C

**(D)**

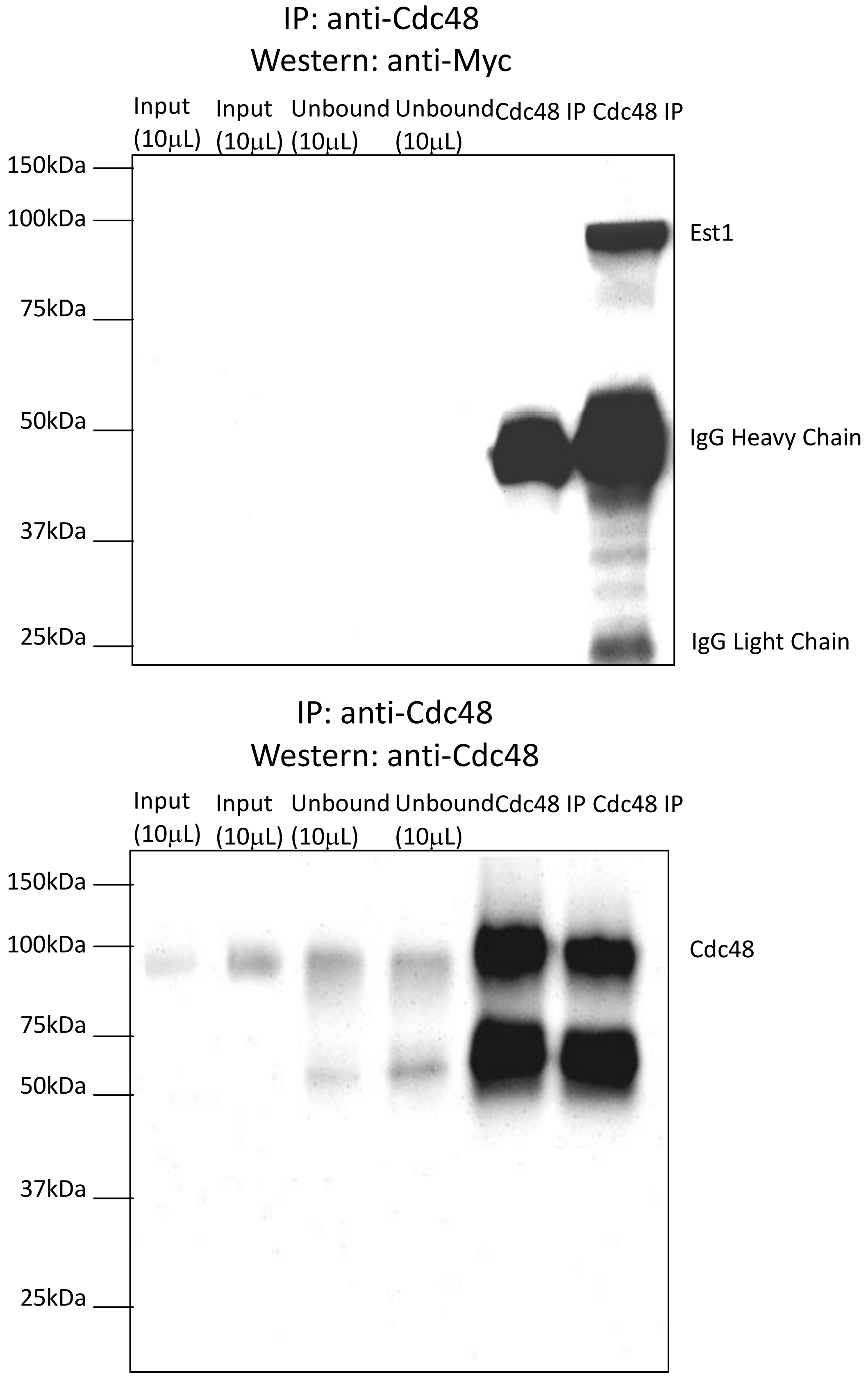
IP: anti-Cdc48
Western: anti-Myc
Input (10μL)
Input (10μL)
Unbound (10μL)
Unbound (10μL)
Cdc48 IP
Cdc48 IP
150kDa
100kDa
75kDa
50kDa
37kDa
25kDa
Est1
IgG Heavy Chain
IgG Light Chain
IP: anti-Cdc48
Western: anti-Cdc48
Input (10μL)
Input (10μL)
Unbound (10μL)
Unbound (10μL)
Cdc48 IP
Cdc48 IP
150kDa
100kDa
75kDa
50kDa
37kDa
25kDa
Cdc48

**Supplementary Figure 4.** Full lane view of (A) Figure 4A, (B) Figure 4B, (C) Figure 4C and (D) Figure 4D.

**Supplementary Figure 5**

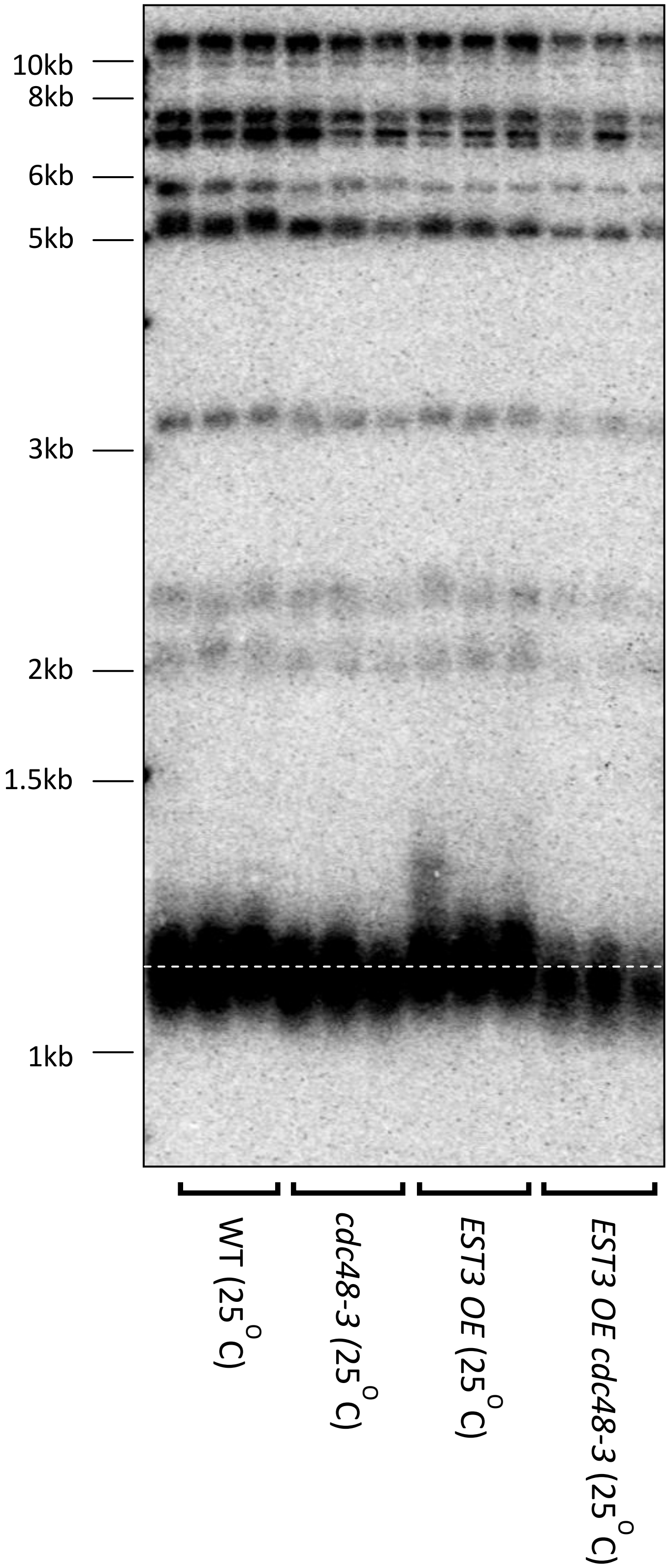

**Supplementary Figure 5.** Over-expression of Est3 in *cdc48-3* cells does not affect telomere length. Southern analysis showed Est3 over-expression does not induce change of telomere length in either *cdc48-3* or WT cells. White dashed line represents the mean telomere length.

**Supplementary Figure 6.**

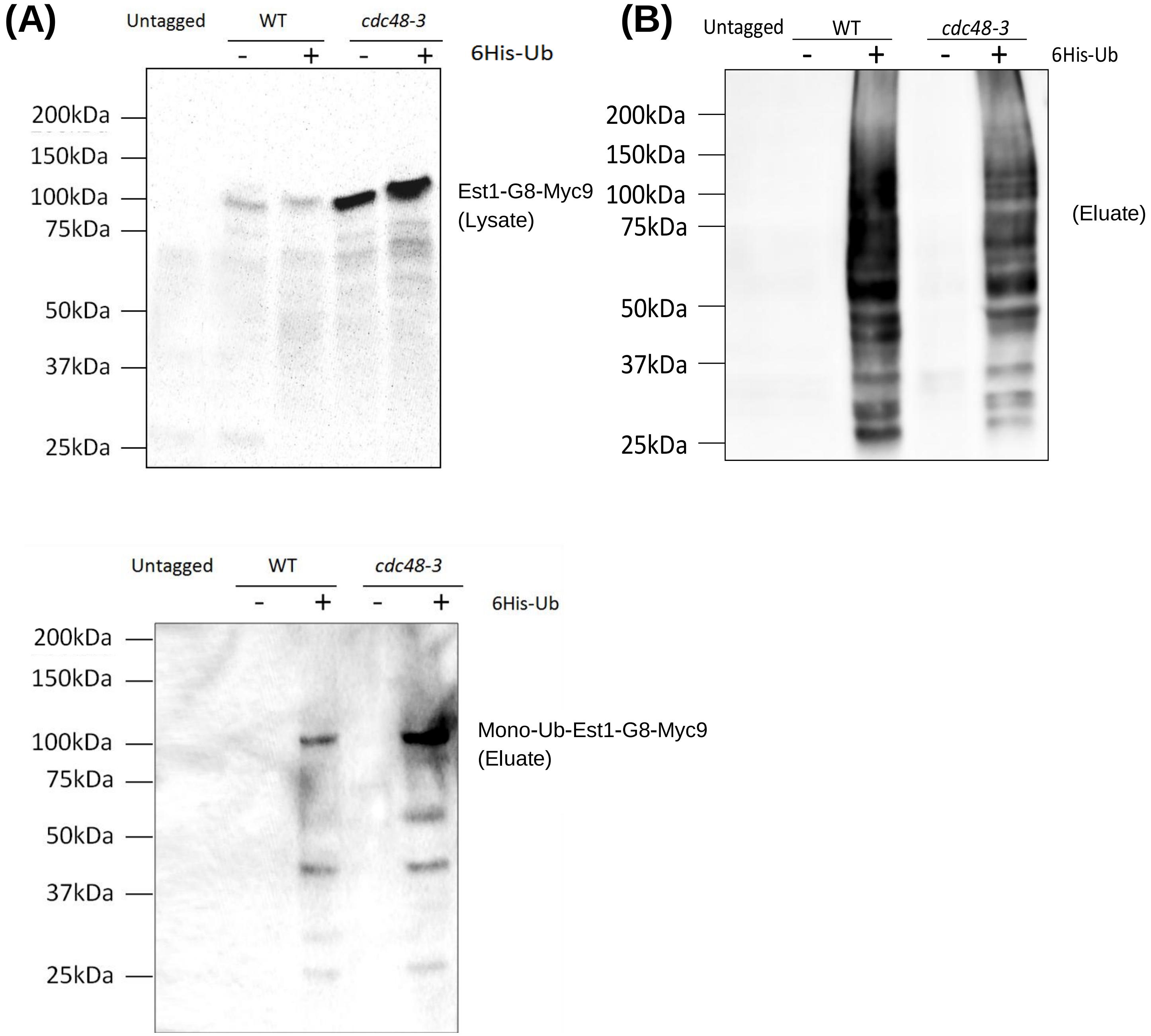

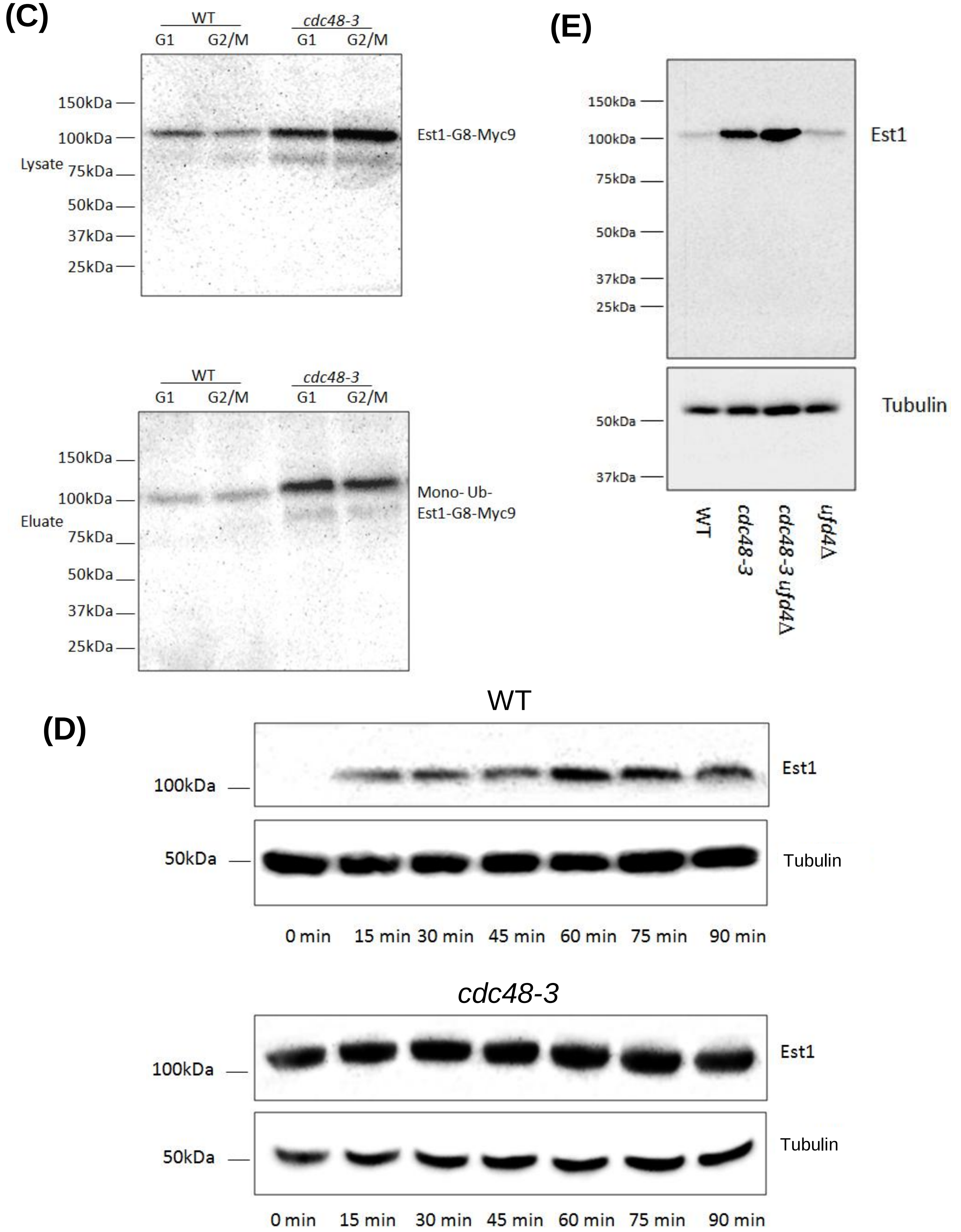

(C)
WT
cdc48-3
G1
G2/M
G1
G2/M
150kDa
100kDa
Lysate
75kDa
50kDa
37kDa
25kDa
Est1-G8-Myc9
WT
cdc48-3
G1
G2/M
G1
G2/M
150kDa
100kDa
Eluate
75kDa
50kDa
37kDa
25kDa
Mono- Ub-
Est1-G8-Myc9
(E)
150kDa
100kDa
75kDa
50kDa
37kDa
25kDa
Est1
50kDa
37kDa
Tubulin
WT
cdc48-3
cdc48-3 ufd4Δ
ufd4Δ
(D)
WT
100kDa
Est1
50kDa
Tubulin
0 min
15 min
30 min
45 min
60 min
75 min
90 min
cdc48-3
100kDa
Est1
50kDa
Tubulin
0 min
15 min
30 min
45 min
60 min
75 min
90 min

**Supplementary Figure 6.** (A) Full lane view of Figure 5A. (B) The efficiency of purification of 6His-Ub (eluate) was controlled by anti-His antibody in Western analysis. (C) Uncropped blots of Figure 5D (D) Full lane view of Figure 5D. (E) Full lane view of Figure 5F.

**Supplementary Figure 7.**

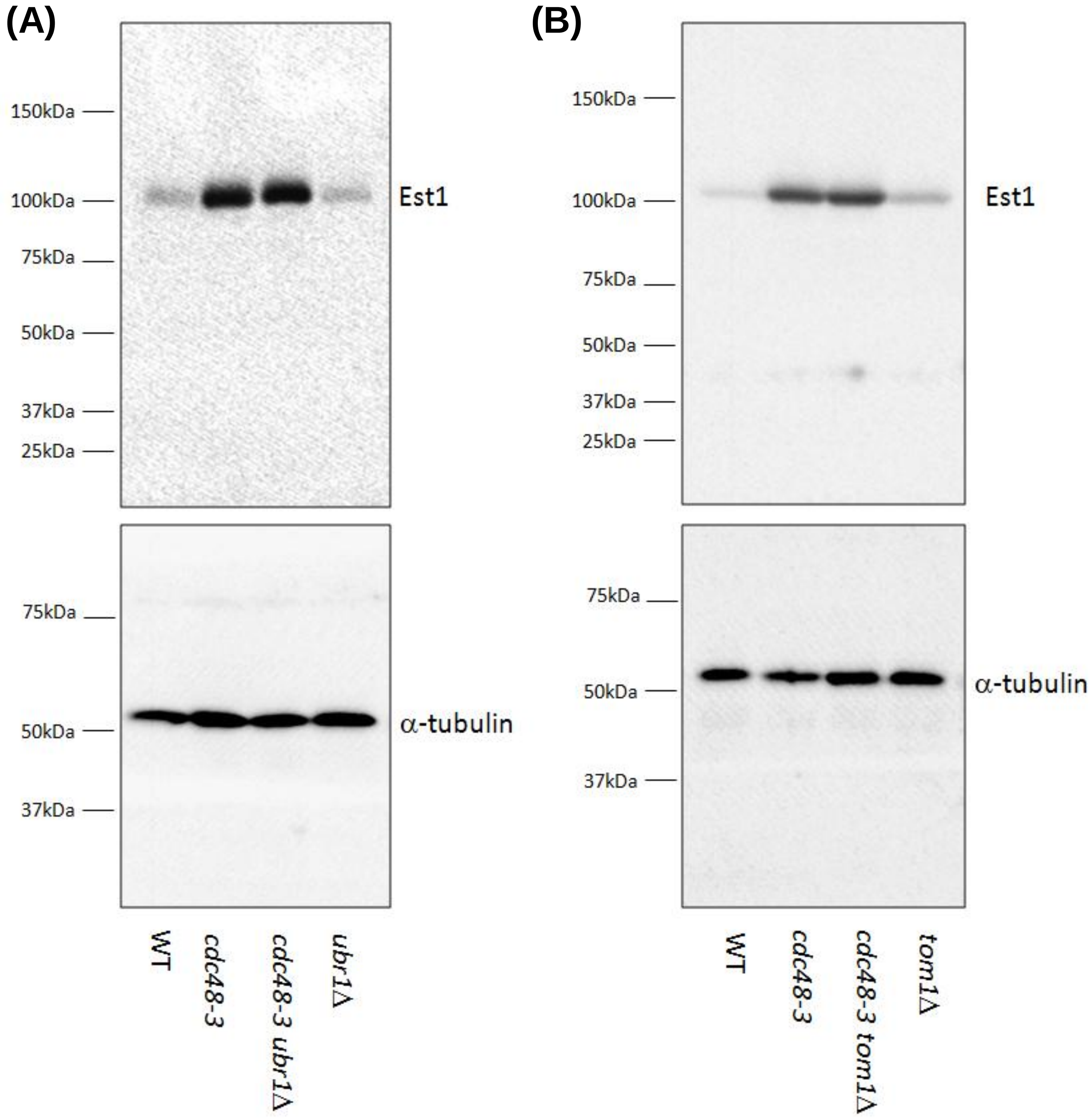

**Supplementary Figure 7. Identification of E3 ubiquitin ligases that affect Est1.** Proteins were prepared from $\alpha$-factor arrested (A) WT, *cdc48-3*, *cdc48-3 ubr1Δ* and *ubr1Δ* cells; (B) WT, *cdc48-3*, *cdc48-3 tom1Δ* and *tom1Δ* cells, grown at 25°C (permissive temperature). Deletion of either *UBR1* or *TOM1* does not affect the expression of Est1 in *cdc48-3* mutant. Est1 was visualized with mouse anti-Myc antibodies and $\alpha$-tubulin was visualized with rat anti-$\alpha$-tubulin antibodies.

**Supplementary Table 1.** Yeast strains and plasmids used in this study.

| Strains | Genotype | References |
|---|---|---|
| W303 (WT) | *MATa leu2-3,112 trp1-1 can1-100 ura3-1 ade2-1 his3-11,15* phi$^{+}$ | 1 |
| YKW001 | W303 *KANMX6-GAL-GFP-EST1 HIS3-GAL-TLC1 HIS3-GAL-GFP-EST2 TRP1-GAL-EST3 bar1Δ::NATMX* | 1 |
| YKW002 | W303 *GAL-GFP-NLS bar1Δ::NATMX* | |
| YKW003 | YKW001 *cdc48-3-HIS3* | |
| RHC677 | W303 *cdc48-3-HIS3* | 2 |
| RHC1126 | W303 *npl4-1* | 2 |
| RHC1122 | W303 *ufd1-2* | 2 |
| YCTT100 | W303 *UTΔU+ Tel VII-L EST1-G8-MYC9-TRP1* | 2 |
| YKW004 | YCTT100 *cdc48-3-HIS3* | |
| YCTT57 | W303 *UTΔU+ Tel VII-L EST2-G8-MYC9-TRP1* | 3 |
| YKW005 | YCTT57 *cdc48-3-HIS3* | |
| YKW006 | W303 *UTΔU+ Tel VII-L KU80-G8-MYC18-TRP1* | 4 |
| YKW007 | YKW006 *cdc48-3-HIS3* | |
| YKW008 | W303 *UTΔU+ Tel VII-L PIF1-MYC13-TRP1* | |
| YKW009 | YKW008 *cdc48-3-HIS3* | |
| YKW010 | W303 *UTΔU+ Tel VII-L CDC13-MYC9-TRP1* | |
| YKW011 | YKW010 *cdc48-3-HIS3* | |
| YKW012 | YCTT100 *npl4-1* | |
| YKW013 | YCTT100 *ufd1-2* | |
| YKW014 | YCTT100 *bar1Δ::NATMX* | |
| YKW015 | YKW004 *bar1Δ::NATMX* | |
| YKW016 | W303 *KANMX6-GAL-GFP-EST1* | |
| YKW017 | YKW016 *cdc48-3-HIS3* | |
| YKW018 | W303 *TRP1-GAL-EST3* | |
| YKW019 | YKW018 *cdc48-3-HIS3* | |

| Plasmids | Description | References |
|---|---|---|
| pRS316-CDC48 | CEN6 URA3 CDC48 | 5 |
| Yep352-6HisUb | 2μ URA3 CUP-6His-Ub | 6 |

## Supplementary References


1 Thomas, B. J. & Rothstein, R. The genetic control of direct-repeat recombination in Saccharomyces: the effect of rad52 and rad1 on mitotic recombination at GAL10, a transcriptionally regulated gene. *Genetics* **123**, 725-738 (1989).

2 Hsieh, M. T. & Chen, R. H. Cdc48 and cofactors Npl4-Ufd1 are important for G1 progression during heat stress by maintaining cell wall integrity in Saccharomyces cerevisiae. *PloS one* **6**, e18988, doi:10.1371/journal.pone.0018988 (2011).

3 Sabourin, M., Tuzon, C. T. & Zakian, V. A. Telomerase and Tel1p preferentially associate with short telomeres in S. cerevisiae. *Molecular cell* **27**, 550-561, doi:10.1016/j.molcel.2007.07.016 (2007).

4 Fisher, T. S., Taggart, A. K. & Zakian, V. A. Cell cycle-dependent regulation of yeast telomerase by Ku. *Nature structural & molecular biology* **11**, 1198-1205, doi:10.1038/nsmb854 (2004).

5 Tran, J. R., Tomsic, L. R. & Brodsky, J. L. A Cdc48p-associated factor modulates endoplasmic reticulum-associated degradation, cell stress, and ubiquitinated protein homeostasis. *The Journal of biological chemistry* **286**, 5744-5755, doi:10.1074/jbc.M110.179259 (2011).

6 Ossareh-Nazari, B. *et al.* Ubiquitylation by the Ltn1 E3 ligase protects 60S ribosomes from starvation-induced selective autophagy. *The Journal of cell biology* **204**, 909-917, doi:10.1083/jcb.201308139 (2014).
